# The combination of the functionalities of feedback circuits is determinant for the attractors’ number and size in pathway-like Boolean networks

**DOI:** 10.1038/srep42023

**Published:** 2017-02-10

**Authors:** Eugenio Azpeitia, Stalin Muñoz, Daniel González-Tokman, Mariana Esther Martínez-Sánchez, Nathan Weinstein, Aurélien Naldi, Elena R. Álvarez-Buylla, David A. Rosenblueth, Luis Mendoza

**Affiliations:** 1 INRIA project-team Virtual Plants, joint with CIRAD and INRA, Montpellier Cedex 5, France; 2 Instituto de Investigaciones en Matemáticas Aplicadas y en Sistemas, Universidad Nacional Autónoma de México, Apdo. 20-126, 01000 México, D.F., México; 3 CONACYT, Instituto de Ecología, A. C., Antiguo camino a Coatepec 351, El Haya, 91070 Xalapa, Veracruz, México; 4 Programa de Doctorado en Ciencias Biomédicas, Universidad Nacional Autónoma de México, México; 5 Instituto de Ecología, Universidad Nacional Autónoma de México, México; 6 ABACUS: Laboratorio de Matemáticas Aplicadas y Cómputo de Alto Rendimiento del Departamento de Matemáticas, Centro de Investigación y de Estudios Avanzados CINVESTAV-IPN, Carretera México-Toluca Km 38.5, La Marquesa, Ocoyoacac, Estado de México, 52740 México; 7 DIMNP UMR CNRS 5235, Université de Montpellier, France; 8 Centro de Ciencias de la Complejidad, Universidad Nacional Autónoma de México, México; 9 Instituto de Investigaciones Biomédicas, Universidad Nacional Autónoma de México, México

## Abstract

Molecular regulation was initially assumed to follow both a unidirectional and a hierarchical organization forming pathways. Regulatory processes, however, form highly interlinked networks with non-hierarchical and non-unidirectional structures that contain statistically overrepresented circuits or motifs. Here, we analyze the behavior of pathways containing non-unidirectional (i.e. bidirectional) and non-hierarchical interactions that create motifs. In comparison with unidirectional and hierarchical pathways, our pathways have a high diversity of behaviors, characterized by the size and number of attractors. Motifs have been studied individually showing that feedback circuit motifs regulate the number and size of attractors. It is less clear what happens in molecular networks that usually contain multiple feedbacks. Here, we find that the way feedback circuits couple to each other (i.e., the combination of the functionalities of feedback circuits) regulate both the number and size of the attractors. We show that the different expected results of epistasis analysis (a method to infer regulatory interactions) are produced by many non-hierarchical and non-unidirectional structures. Thus, these structures cannot be correctly inferred by epistasis analysis. Finally, we show that the combinations of functionalities, combined with other network properties, allow for a better characterization of regulatory structures.

Early approaches considered that molecular regulation is composed of hierarchical and unidirectional interactions, where “upstream” molecules regulate “downstream” molecules, but molecules are not regulated by molecules at the same or lower levels (molecules usually represent genes and gene products^1^). Unidirectional and hierarchical interactions form pathways, comprised by an input, internal molecules, and an output. Pathway dynamics (i.e., how the components in the pathway are activated and inhibited in time), follow a sequential order of regulatory events going from the input to the output through the internal molecules. Even though pathway dynamics seem to be an inherent property of molecular regulation^2^,^3^, molecular regulation is usually complex, forming highly inter-connected networks that are neither unidirectional nor hierarchical^4^,^5^,^6^. Our objective is to systematically study the effect of including non-hierarchical and non-unidirectional (i.e. bidirectional) interactions within pathways.

Adding interactions within a pathway modifies the pathway structure (i.e., the interaction graph describing the regulatory interactions between the molecules), allowing for the appearance of regulatory motifs. Motifs are statistically overrepresented regulatory interactions found in molecular networks^5^. Among the motifs, circular chains of oriented interactions known as feedback circuits, are specially relevant, because they regulate both the number and size of the attractors^7^,^8^. Attractors are stationary network states that represent biologically meaningful properties^9^, such as cell identity^10^,^11^. Positive feedback circuits are necessary to have multiple attractors and negative feedback circuits are necessary to produce cyclic attractors^12^,^13^. Moreover, the maximum possible number of attractors is regulated by positive feedback circuits^14^. It is not completely clear, however, how the precise size and number of attractors are regulated within molecular networks where many feedback circuits are present^15^,^16^,^17^. In this work, we focus on the effect that adding non-hierarchical and non-unidirectional interactions has over the size and the number of attractors. In particular, we study how multiple feedback circuits, that result from the added interactions, regulate the size and number of attractors.

The study of the properties of non-hierarchical and non-unidirectional networks is fundamental to detect and solve limitations of traditional analyses. In particular, experimental research at small scales commonly uses traditional analyses that rely on the hierarchical and unidirectional assumptions. For example, assuming that regulatory interactions are hierarchical and unidirectional, epistasis analysis, as described in refs 1,18,19, can distinguish between different pathway structures by interpreting and organizing epistatic interactions (sensu Bateson^20^). The presence of non-unidirectional and non-hierarchical interactions, however, can produce incomplete or even wrong inferences when using epistasis analysis^21^. Here, we look for useful traits to distinguish between non-hierarchical and non-unidirectional regulatory structures that could improve inference methods, such as epistasis analysis.

In this work, we use synchronous Boolean networks to do a comprehensive analysis of Pathway-like networks (PLNs) that are non-hierarchical and non-unidirectional versions of pathways. We focus on the dynamical properties of PLNs, represented by both the number and the size of attractors. We show that PLNs have a large dynamical diversity in comparison with pathways. We confirm that feedback circuits are important regulators of the size and the number of attractors in a network. Then, we show for the first time, as far as we know, that the precise size and number of attractors in networks with multiple feedback circuits is in large part determined by the combination of the functionalities of feedback circuits. This combination refers to the network states where the feedback circuits in a network have an actual effect over the network dynamics. Then, we show that there are a vast number of PLNs producing the same set of attractors, hindering a correct inference of PLNs structures with methods such as epistasis analysis. Interestingly, PLNs producing the same set of attractors also have remarkably similar sets of combinations of functionalities. Thus, we explore how to use the combination of functionalities combined with both the network dynamic and structure for the characterization of PLNs. Our results show that PLNs with the same structure and with the same combination of functionality form small regions with dynamically distinguishable properties. Thus, the study of such regions could facilitate the inference of such networks.

## Methods

### Boolean networks

In this work, we use the Boolean formalism to model molecular networks for the following reasons. (1) Because of their simplicity, Boolean networks are well suited to perform analyses in a large number of networks, without needing to deal with problems such as parameter estimation. (2) Despite their simplicity, Boolean networks obtain biologically meaningful results (e.g., refs 10, 11 and 22, 23, 24). (3) Feedback circuits are fundamental for this work, and feedback circuit functionality is well studied in Boolean networks^25^,^26^. (4) Here we will focus on the size and number of attractors, and it has been proven that in Boolean networks positive and negative feedback circuits are a necessary condition for multistability and oscillations, respectively^13^,^27^. (5) We will use epistasis analysis, as described in refs 1, 18 and 19, that assumes that the molecules behave as Boolean variables. Hence Boolean networks are a natural and simple extension of epistasis analysis.

Molecular networks have variables representing the molecules included in the network (e.g., genes, proteins, hormones, among other molecules). In Boolean networks, variables can only take one of two possible values, 0 or 1, and their dynamics is described by


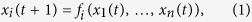


where *x_i_(t* + 1) represents the value of variable i at the time *t* + 1 as a Boolean function *F_i_* of its n regulators *x*_*i*_(*t*), ..., *x*_*n*_(*t*) at the current time. In particular, we use synchronous Boolean networks, where the value of all variables is updated at each time step.

The set of all values of the variables at time t is a network state. The number of network states in any network is equal to 2^*v*^, where *v* is the number of variables. Stationary network states are known as attractors. Single-state, stationary configurations are known as fixed-point attractors, whereas a set of network states that orderly repeat correspond to cyclic attractors. The size of an attractor is equal to the number of network states that conform such attractor.

**Figure 1:**
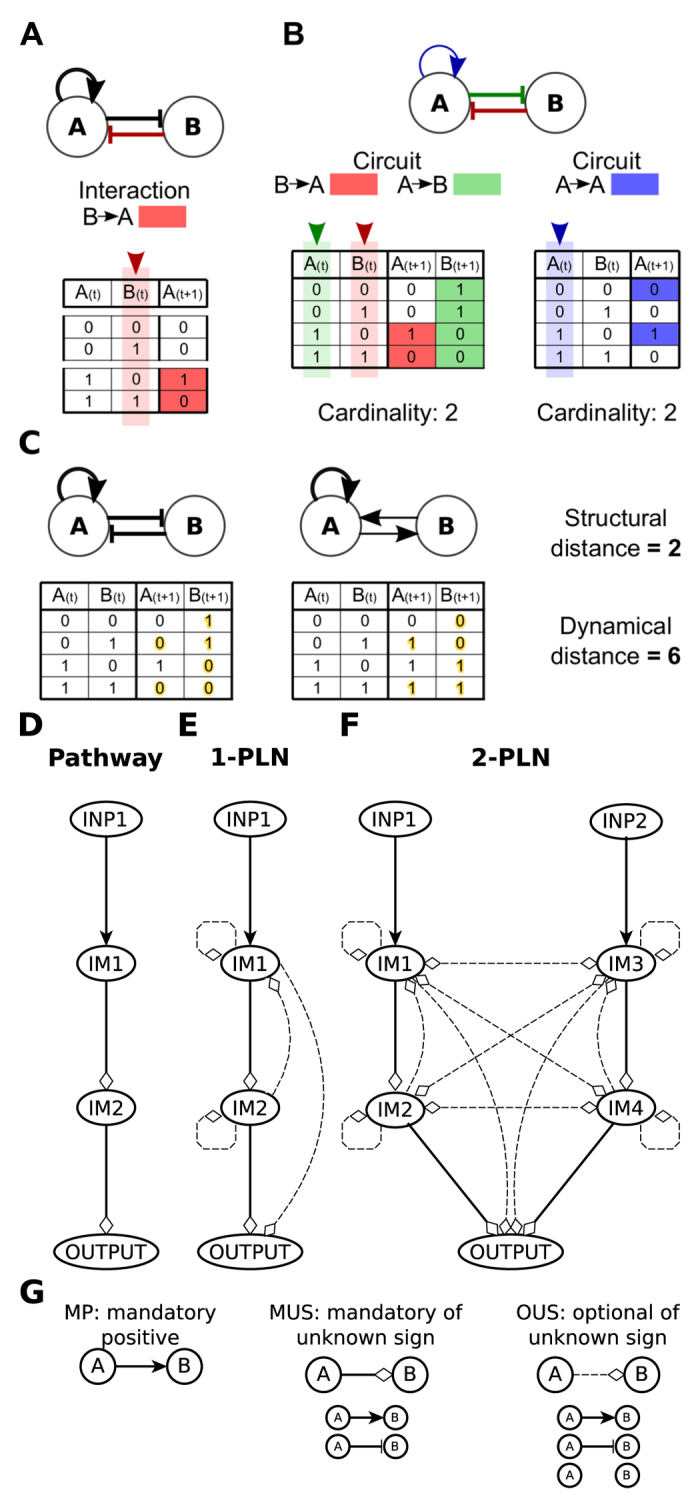
Basic concepts and Pathway-like networks. (**A**) Analysis of the functionality of the interaction from *B* to *A* (red link in the interaction graph above). Notice that in the last pair of lines of the table, *A(t* + 1) and *B(t*) values are opposite, while *A(t*) is equal to 1 in both lines, indicating that a change, only in the value of *B* (highlighted in light red) can change the value of *A* in the next time step (highlighted in red). Thus, the interaction is functional and negative. (**B**) Above, the interaction graph of a network containing two positive feedback circuits, one between *A* and *B* (red and green interactions) and the other of *A* with itself (blue interaction). As observed below, both feedback circuits are functional, as there exists at least one network state (the network states are conformed by all the combination of *A(t*) and *B(t*) values in the left side of the tables) where all the interactions of each circuit are functional. The cardinality is equal to the sum of the network states where the feedback circuit is functional. Thus, the combination of functionalities of this network comprises a positive feedback circuit from *A* to *A* with a cardinality of two, and a positive feedback circuit between *A* and *B* with a cardinality of two. (**C**) Dynamical and structural distance between two networks. Above, the interaction graphs of two different networks. The structural distance is equal to the differences between the interaction graphs of the networks. Below, the state diagrams of the networks. The dynamical distance is equal to the sum of the differences (highlighted in yellow) between the state diagrams of both networks. (**D**) Pathway, (**E**) 1-PLN and (**F**) 2-PLN structure. INP = INPUT and IM = Intermediary Molecules. (**G**) Interaction types considered in this work to construct pathways, 1-PLNs and 2-PLNs: mandatory positive (MP), mandatory of unknown sign (MUS) and optional of unknown sign (OUS). Mandatory interactions are always present, while optional interactions can be either present or not. Positive interactions are always positive. Unknown sign interactions can be either positive or negative.

For this work it is important to note that not all interactions in a network structure are necessarily functional. An interaction from a variable *i* to a variable *j* is considered functional, if *j* can change its value due to a change only in the value of *i*. The interaction sign is positive, if the change in the value of *j* goes in the same direction as the change in the value of *i*, and is negative otherwise (Fig. 1A; see Supplementary Methods for formal definitions). Note that a variable can act as a positive regulator and as a negative regulator of the same variable in different network states. According to some authors, interactions where a variable is a positive and negative regulator of another variable are not common in real molecular networks^21^,^28^,^29^,^30^. On the other hand, non-functional interactions, where a regulator does not influence the value of the regulated variable, do not provide any meaningful information. Hence, in the networks analyzed here, we forbid both non-functional interactions and interactions where a regulatory variable has both a positive and a negative influence over another variable.

A feedback circuit is a set of directed interactions forming a closed path. Feedback circuits can be positive or negative. The sign of a feedback circuit is given by the signs of its interactions. A circuit is positive if it has an even number of negative interactions, it is negative otherwise. The sole presence of a circuit in a network does not guarantee its functionality. Thus, a circuit is considered functional if all interactions of the circuit are functional in a set of common network states^25^. Notice that using our sign and functionality definitions, there are four categories of feedback circuits, positive functional, negative functional, positive non-functional and negative non-functional. Furthermore, in this work, functional feedback circuits are characterized by the sign and number of network states where the circuit is functional (cardinality). The combination of the functionalities of feedback circuits is the set of all circuits functionalities present in a given network (Fig. 1B).

Here, we compare the structural and dynamical distance between networks. The structural distance is the sum of connectivity differences between the interaction graphs of two different networks. In particular, we considered as a connectivity difference any difference in the number or the sign of the interactions of two interaction graphs (Fig. 1C). The dynamic of a model can be visualized as a state diagram. A state diagram is a directed graph where each state in the state space represents a network state and is connected to the update state reached after applying to it the Boolean functions associated with the variables. The dynamic distance is a measure of the difference between the state diagrams of two networks. To calculate the dynamic distance we sum the differences in the states reached after applying to each network state the Boolean functions of each network (Fig. 1C).

### Pathway-like networks construction

We analyze two types of networks, namely pathways and pathway-like networks (PLNs), both containing different types of interactions identified with the acronyms MUS, OUS and MP, which stand for mandatory unknown sign, optional unknown sign, and mandatory positive, respectively (Fig. 1G). MUS and MP interactions form unidirectional and hierarchical pathway structures (Fig. 1D). PLNs contain MUS, MP and OUS interactions (Fig. 1E,F). We construct two PLNs variants: single (1-PLNs) and double (2-PLNs). 1-PLNs is the set of networks that contains a pathway within its structure and at least one OUS interaction (Fig. 1E). 2-PLNs is the set of networks composed by two parallel pathways regulating the same output and at least one OUS interaction (Fig. 1F). 2-PLNs may have cross-regulation between their constituent pathways, which is a common biological situation.

We used two different approximations to analyze the size and the number of attractors. (1) We simulated each PLN starting from all network states until finding the attractors. (2) We use symbolic algorithms to search for PLNs with a specific number and size of attractors. It is important to note that in most cases, a given network structure can be described by more than one set of Boolean functions. Thus, there is a vast number possible PLNs structural and dynamical variants. Without considering the sign of the interactions, the number of possible structures for a network with v variables is equal to 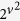. The Boolean functions associated with a variable is 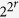. where *r* is the number of regulators of the variable. Therefore, the total number of possible Boolean functions for a completely interconnected network with v variables is 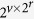. In particular, there are ≈4.15 × 10^34^ 2-PLNs. The analysis of such a number of variants is humanly and computationally unfeasible. Thus, we use random sampling or constrained the maximum number of interactions for 2-PLNs analyses (see Supplementary Methods for more details).

## Results

### Non-hierarchical and non-unidirectional interactions greatly increases the dynamical diversity of pathways

Conventionally, molecular regulation is represented as unidirectional and hierarchical pathways, comprised by an input, internal molecules, and an output (Fig. 1D). To study pathways with more realistic structures, we consider the possibility that the internal molecules may regulate any component inside the pathway (excluding the input). We call this structure a Pathway-like network (PLN). We analyzed single (1-PLNs) and double (2-PLNs) PLNs (Fig. 1E,F). PLNs have non-unidirectional interactions, and the internal molecules are non-hierarchically organized. For simplicity, we refer to PLNs as non-unidirectional and non-hierarchical structures. Regarding their dynamics, PLNs are constructed in such a way that the generation of Boolean functions producing “meaningless” behaviors is forbidden^21^,^28^. Altogether, PLNs contain realistic structural and dynamical properties (see Methods).

**Figure 2:**
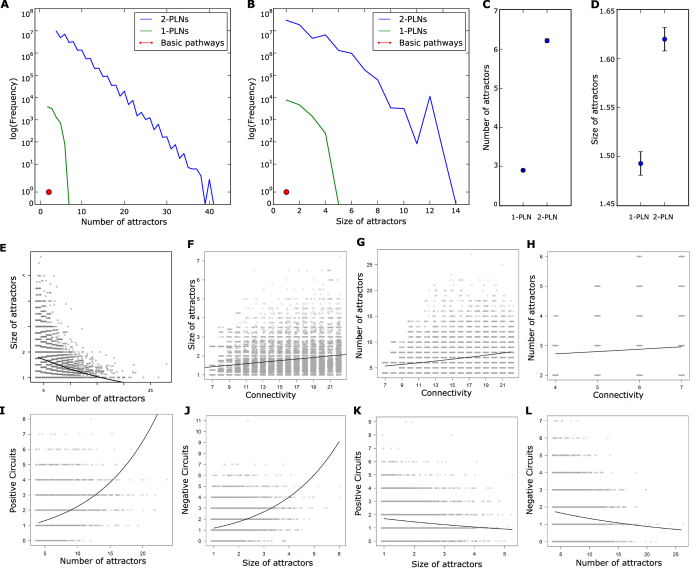
PLNs properties and relations. Distribution of the (**A**) number and (**B**) size of the attractors in pathways, 1-PLNs and 2-PLNs. Mean and confidence interval of the (**C**) mean number and (**D**) mean size of attractors for 1-PLN and 2-PLNs. (**E**) Relation between the number and mean size of attractors of 2-PLNs. (**F**) Mean size of attractors vs. 2-PLNs connectivity. Number of attractors vs. connectivity of 2-PLNs (**G**) and 1-PLNs (**H**). 2-PLNs mean size of attractors vs. quantity of (**I**) positive and (**J**) negative feedback circuits, respectively. 2-PLNs number of attractors vs. quantity of (**K**) positive and (**L**) negative feedback circuits, respectively. In E-L each point represents a single 2-PLN or 1-PLN data, while the line represents the values predicted by Poisson GLM. Points in are displaced the X axis only for visual purpose. Similar results were found in 1-PLNs (Fig. S1).

We analyzed the dynamical diversity, measured as the number and the size of attractors in pathways, 1-PLNs and 2-PLNs. The simulation of all pathways, all 1-PLNs and more than randomly selected 30 million 2-PLNs. showed that the dynamical diversity vastly increases from pathways to 1-PLNs to 2-PLNs (Fig. 2A,B). Because the inputs follow the identity function, the minimum number of attractors is equal to 2|inputsl|, where *inputs* is the set of inputs. On the other hand, the maximum number of attractors found in our simulations are 2, 6, and 40 in pathways, 1-PLNs and 2-PLNs, respectively. This increase is also observed in the mean values, where the mean value of the number of attractors of 2-PLNs is significantly larger than in 1-PLNs (*P* < 0.001; Fig. 2C; see Supplementary information file for detailed information about all statistical results). Similar results can be observed for the size of attractors. Specifically, the maximum sizes are 1, 4, and 13 for pathways, 1-PLNs and 2-PLNs, respectively. Here, too, the mean value of the size of the 2-PLNs attractors is significantly larger (*P* < 0.001; Fig. 2D). Note that both the mean size and mean number of attractors are much closer to their minimum value than to their maximum value. This indicates that most networks have a small number of attractors of small size; and at first sight it seems that they might fit a long-tailed distribution. However, these data do not fit power-law, logarithmic, exponential, normal or Poisson distributions. Nevertheless, our results clearly show that the overall diversity of dynamical behaviors grows from pathways to 1-PLNs to 2-PLNs, due to the addition of non-unidirectional and non-hierarchical interactions.

### Feedback circuits’ role in the regulation of the PLNs attractors properties

We noticed that there is a statistically significant negative relation between the mean size and the number of attractors (*P* < 0.001; Figs 2E and S1A), suggesting that both the size and the number of attractors are regulated by the same property, but in opposite directions. Then, we noticed that as we increase the number of non-unidirectional and non-hierarchical interactions, the size of the attractors increases in 2-PLNs (*P* < 0.001; Fig. 2F) and the number of attractors increases in both 1-PLNs and 2-PLNs (*P* < 0.001; Fig. 2G,H), showing that the property regulating the size and number of attractors, increases as we increase the connectivity of the PLNs. Positive feedback circuits are necessary for multistability, while negative feedback circuits are necessary to produce cyclic attractors^12^,^13^ and adding more non-unidirectional and non-hierarchical interactions creates more feedback circuits. Thus, it seems reasonable to think that the appearance of feedback circuits in PLNs structures is responsible for the increase in the dynamical diversity, and that depending on their sign, the feedback circuits will increase the size or the number of attractors. Indeed, positive feedback circuits are positively related with the number of attractors (P < 0.001), and negative feedback circuits are positively related with the size of attractors (*P* < 0.001;; Fig. 2I,J). Furthermore, positive feedback circuits have a negative relation with the size of attractors (*P* < 0.001), while negative feedback circuits have a negative relation with the number of attractors (*P* < 0.001; Figs 2K,L and S1B–E), explaining the negative relation between number and size.

**Figure 3:**
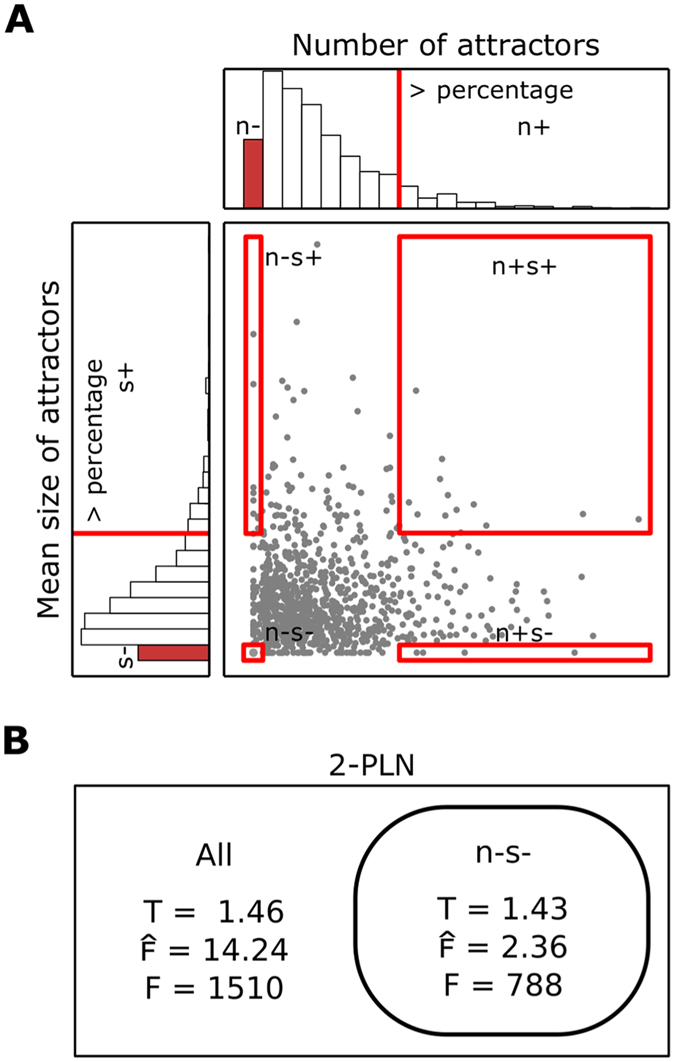
PLNs attractors properties regulation by feedback circuits. (**A**) Classification of PLNs according to the number and mean size of the attractors: *n*+ more attractors than 70%, 80%, 90%, 95% or 99% of the PLNs, *n*− minimum number of attractors, *s*+ mean size of attractors bigger than 70%, 80%, 90%, 95% or 99% of the PLNs and *s*− fixed-point attractors. (**B**) Total number of combinations of functionalities in 50 random sampling of 2,000 2-PLNs and 2,000 n−s− 2-PLNs (F). Mean number of combinations of functionalities contained in 2-PLNs structures and in n−s− 2-PLNs structures obtained from a sampling of one million 2-PLNs randomly generated (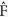). Mean number of 2-PLNs and n−s− 2-PLNs structures that contained each combination of functionalies obtained from a sampling of one million 2-PLNs randomly generated (T).

To better analyze how feedback circuits regulate the size and number of attractors we divided PLNs in four categories. (1) PLNs with the minimum number of attractors, whose mean size is bigger than the mean size of certain percentage of the total set of PLNs analyzed (n−s+). (2) PLNs with more attractors than certain percentage of the PLNs, all fixed-point attractors (n+s−). (3) PLNs with the minimum number attractors, all fixed-point (n−s−). (4) PLNs with more attractors than a percentage of the PLNs, whose size is bigger than the same percentage of the PLNs (n+s+) (Fig. 3A). We classified all 1-PLNs and selected 10,000 2-PLNs of each category from a sample of over six million 2-PLNs that were randomly generated. We used five percentages for the size and the number of attractors, namely 70%, 80%, 90%, 95% and 99%. In 1-PLNs it is not possible to define 99% and 70%, due to the limited amount of data, and we did not find n+s+ 1-PLNs for any percentage and for n+s+ 2-PLNs using 90% or higher. Then, to see if the quantity of feedback circuits in a PLN is important to determine the number and the size of the attractors, we compared the total number of feedback circuits of n+s+, n+s−, n−s+ and n−s−. In 2-PLNs, the total number of feedback circuits consistently and significantly increase from n−s− to n−s+ to n+s− to n+s+ (Tables 1 and S1), suggesting that as the number of feedback circuits increases, the number and size of attractors tends to increases too. According to Kwon *et al*.^31^, the positive/negative feedback circuits ratio provides the trend of the number and the size of attractors, with larger ratio values for networks with more attractors of smaller size. If this is the case, n+s− PLNs should have a bigger positive/negative feedback circuits ratio than n−s+. On the other hand, n−s− and n+s+ could have a balance between positive and negative feedback circuits. As observed in Tables 1, 2, S1 and S2, this is indeed the case in both 1-PLNs and 2-PLNs using all percentages (*P* < 0.001). From the circuits ratio result, we can expect the PLNs n+s−to have more positive feedback circuits and fewer negative feedback circuits, the opposite behavior for n−s+, and a similar number of positive and negative feedback circuits in the n+s+ and n−s−. As observed, this is the case in both 1-PLNs or 2-PLNs (Tables 1 and 2). Thus, our results support the idea that the positive/negative feedback circuits ratio provides the correct trend for the size and the number of attractors.

### The combination of the functionalities of the feedback circuits regulates the attractors size and number

It is important to note that because the feedback circuits ratio provides a statistical relation of how the number and size of attractors will behave, there could be some instances where the size and number are not consistent with the feedback circuits ratio. In fact, we detected PLNs that behaved opposite to what was expected from their feedback circuits ratio. For example, we found PLNs n+s− with a lower positive/negative feedback circuits ratio than the mean ratio in PLNs n−s+ and vice versa (e.g., Supplementary Fig. S2). Observe that the mere presence of a feedback circuit in the structure does not guarantee the expected behavior unless the circuit is functional^8^,^25^. Then, the feedback circuits ratio might not predict the correct trend when some of the circuits are not functional. For a circuit to be functional, all interactions within such a circuit should be functional in a shared set of network states^25^ (Fig. 1B). To study whether the functionality of the circuits is behind the unexpected behaviors, we analyzed the functionality of feedback circuits of all 1-PLNs and a sampling of one million 2-PLNs. Indeed, we found that not all feedback circuits in the PLNs are functional. However, considering only functional feedback circuits do not eliminated all the unexpected behaviors (e.g., Supplementary Fig. S2). This result indicates that the circuits’ ratio and functionality is not enough to understand how the size and the number of attractors are regulated. Thus, we decided to look for complementary properties that could allow us to better understand how the number and size of attractors are regulated.

We noticed that the effect the multiple feedback circuits have over the number and size of the attractors depends, not only on their sign and functionality, but also on how circuits couple to each other. In particular, we observed that the same feedback circuits in the same network structure can be functional in a different number of network states (i.e., a circuit can have different cardinalities) and changes in the cardinality of a circuit can (1) modify the number and the size of the attractors and (2) modify the cardinality of other circuits (Fig. S3). Thus, the cardinality of the circuits could be important to understand how multiple feedback circuits couple and determine the number and size of attractors. Accordingly, we defined the combinations of the functionalities of feedback circuits (hereinafter named combinations of functionalities) of a PLNs as the set of all its circuits functionalities, characterized by sign and cardinality (Fig. 1B). If a specific number and size of attractors is produced only by certain combinations of functionalities, a set of PLNs with the same number and size of attractors, such as n−s− PLNs, should have a reduced number of combinations of functionalities than a set of PLNs with different number and size of attractors. Thus, to study if only certain combinations of functionalities can produce a specific number and size of attractors, we compared the number of combination of functionalities in (1) all 1-PLNs against all n−s− 1-PLNs and in (2) 50 samples of 2,000 2-PLNs against 2,000 n−s− 2-PLN, randomly selected from 50 samples of 100,000 2-PLNs randomly generated. Indeed, there are only eight combinations of functionalities in n−s− 1-PLNs and 62 in 1-PLNs. Similarly, there are significantly fewer combinations of functionalities in n−s− 2-PLNs (788 ± 7.74) than in 2-PLNs (1,510 ± 11.07; *P* < 0.001; Fig. 3B). This result supports the idea that specific number and size of attractors are produced by specific combinations of functionalities. Then, if only certain combinations of functionalities are capable of produce a specific number and size of attractors, only some of the combinations of functionalities contained in a structure should produce attractors of a specific size and number. We found that by selecting the n−s− PLNs, the mean number of combinations of functionalities contained in each structure in all 1-PLNs and in one million randomly selected 2-PLNs diminished from 8.85 ± 21.38 to 2.66 ± 3.65 and from 14.24 ± 0.199 to 2.36 ± 0.146 in 1-PLNs and 2-PLNs, respectively (Fig. 3B). This reduction is significant in the 2-PLNs case (*P* < 0.001), demonstrating that over the large number of existing combinations of functionalities in a structure, only some of them are able to produce a specific number and size of attractors.

Observe that networks with different structures can produce the same combination of functionalities (Fig. S3A,B). If the combination of functionalities regulates the attractors’ size and number, different PLN structures producing the same combination of functionalities should also be able to produce the same number and size of attractors. The latter would be better supported if each combination of functionalities were, on average, contained in the same number of structures in a set of PLNs with the same size and number of attractors and in a set of PLNs with different size and number of attractors. To analyze if this is the case, we compared the mean number structures containing each combination of functionalities in (1) all 1-PLNs structures against all n−s− 1-PLN structures and in (2) the structures 2-PLNs against the structures of n−s− 2-PLN, from a sample of one million randomly selected 2-PLNs. Each combination of functionalities is contained in exactly two structures in both n−s− 1-PLNs and the complete set of 1-PLNs. Similarly, the combinations of functionalities of 2-PLNs and n−s− 2-PLNs are contained in 1.46 ± 0.005 and in 1.43 ± 0.0022 structures (Fig. 3B), respectively, showing no significant differences (*P* < 0.331). By doing an analysis of individual PLNs, we find some instances, in which the same combination of functionality produce a different number or size of attractors. We looked for, but did not find, other complementary properties, such as additional interactions, that allows to completely understand how the number and the size of the attractors is regulated. However, our results strongly suggest that network structures with the same number of variables and with the same combination of functionalities, in general, can produce the same number and size of attractors.

### Combinations of the functionalities of feedback circuits and the analysis of epistasis

It is important to note that our definition of combination of functionalities is independent of the specific network states that are attractors. Thus, a complementary way to test if the combinations of functionalities regulate the number and the size of attractors, is to search for networks with the same number of attractors of the same size, but whose attractors are represented by different network states. These networks should be produced by the same or similar sets of combinations of functionalities.

**Figure 4:**
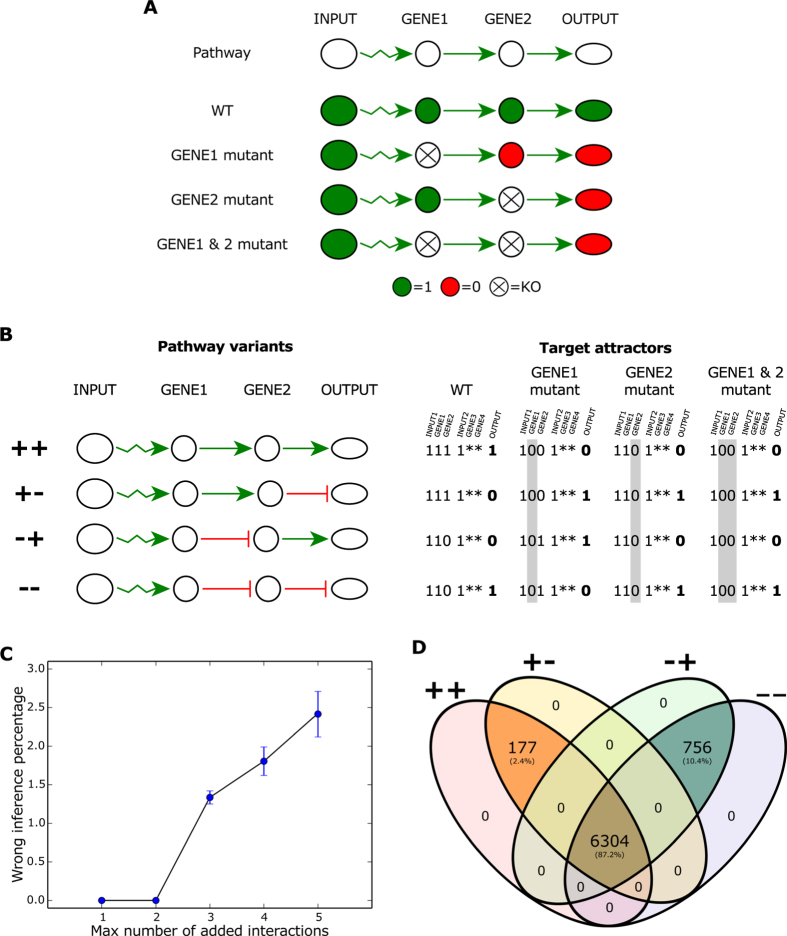
Characterization of the PLNs that produce the epistasis results. (**A**) Boolean interpretation of the epistasis analysis using the + + pathway variant shown in (**B**). (**B**) Target attractors for each of the four pathway variants using the epistasis analysis. Mutated genes are highlighted in grey. For 1-PLNs we only consider INP1, GENE1/2 and OUTPUT values. Epistasis analysis do not consider the possibility of a second parallel and cross-talking pathway. Thus, the asterisks represent unknown values for GENE3 and GENE4 in the 2-PLNs case. Input values are considered equal to 1 because the epistasis analysis assumes that the input values are constant during the experiment^1^. (**C**) Percentage of 2-PLNs that produce the target attractors of a different pathway variant than the pathway variant contained in the 2-PLN as we added more interactions to the unidirectional and hierarchical pathway structure. (**D**) Percentage of combinations of functionalities shared between the PLNs that produced each of the four sets of target attractors.

We noticed that epistasis analysis, as described by refs 1, 18 and 19, provides this possibility. Briefly, epistasis is a term used when the phenotype of an allele is masked by an allele in another locus. The gene with the allele whose phenotype persists when the alleles of both loci are present is called epistatic gene, while the other is the hypostatic gene. Epistasis analysis uses a simple set of two rules to order the epistatic and hypostatic genes^1^,^18^,^19^. First, in a double-mutant experiment, the epistatic gene is upstream and positively regulates the downstream gene when the two genes used in the double-mutant display a characteristic single-mutant phenotype under the same condition. And second, in a double mutant experiment, the epistatic gene is downstream and is negatively regulated by the upstream gene when the two genes display a characteristic single mutant phenotype under different conditions. The epistasis analysis can be formalised in Boolean terms in a straightforward way (Fig. 4A). Observe that the possible combinations of positive and negative interactions in a pathway already gives four pathway variants that have the same number and size of attractors, but the attractors of each pathway variant correspond to different network states. Previous works had characterized the expected results of the four pathway variant^1^,^18^,^19^. Thus, we used the expected results of each pathway variant by the epistasis analysis to further analyze the importance of the combinations of functionalities in the regulation of the size and number of attractors.We named each pathway variant according to the sign of the regulation from GENE1 to GENE2 and from GENE2 to the OUTPUT, as ++, +−, −+ and − −. Then, we interpreted the epistasis analysis expected results for each pathway variant as the set of target attractors in our PLNs (Fig. 4B) and analyzed the number of 1-PLNs and 2-PLNs that achieved each of the four possible sets of target attractors.

The number of PLNs able to produce the target attractors in the 1-PLNs is 60 for the ++ and +− variants and 68 for the −+ and −− variants. We were unable to perform an exhaustive 2-PLNs search because of the astronomical number of redundant 2-PLNs found. Restricting our search to 2-PLNs with a maximum of five extra interactions compared to the pathway structure, we found more than 4.731 × 10^7^ 2-PLNs that produced each set of target attractors. In the 1-PLNs case, the target attractors of a pathway variant are only obtained when the same pathway variant is contained in the PLN. On the other hand, ≈2.41% of the 2-PLNs produced wrong inferences. We considered PLNs producing the target attractors of a pathway variant different from the pathway variant contained in its structure as wrong inferences. This demonstrates that epistasis analysis can produce wrong regulatory inferences when analyzing non-unidirectional and non-hierarchical networks, as stated before by our group^21^. The wrong inferences increase as we added more interactions (Fig. 4C) and are produced thanks to the added interactions between the parallel pathways contained in the 2-PLNs. It is interesting to note that, apparently, when there are wrong inferences, the extra interactions produce an alternative pathway that conformed with the expected pathway variant but that contained some intermediary steps between GENE1/2 or between GENE2 and OUTPUT (see some examples in Fig. S4). However, the alternative pathway by itself is not sufficient to produce the expected results as the alternative pathway can be created by adding one interaction and inconsistencies between the target attractors and the pathway variant contained in the PLNs appear only when we added three or more interactions (Fig. 4C). Thus, our results demonstrate that epistasis analysis produce wrong and incomplete inferences in non-hierarchical and non-unidirectional networks, but when a network has a low connectivity, wrong regulatory inferences are scarce.

The combinations of functionalities that produced the four target attractors are almost the same in both 1-PLNs and 2-PLNs. In 1-PLNs all four target attractors share one combination of functionalities (see Fig. S5). This is a meaningful result as that is the only combination of functionalities that produced the target attractors of the variants ++ and +− and one of the two combination of functionalities that produce the target attractors of the variants −+ and −−. In the 2-PLNs case, there are 6,481 combinations of functionalities that produced the target attractors of the variants ++ and +− and 7,060 that produced the target attractors of the variants −+ and −−. 6,304 combinations functionalities are shared by the four pathway variant (Fig. 4D). This is an astonishing result that greatly supports the importance of the combinations of functionalities to determine the attractors properties, as the target attractors vary for each pathway variant, indicating that the common feature among the PLNs found are the number and the size of attractors.

### Comprehensive characterization of PLNs

Having multiple networks producing the same set of results raises a problem for the analyses of molecular regulation by methods, such as the epistasis analysis, as they cannot distinguish between these networks. Hence, we did an exploration of which properties could be useful to distinguish between these networks. For this characterization, we used all 1-PLNs and 2-PLNs with no more than two interactions added to the unidirectional and hierarchical pathway structure, because comparison of 2-PLNs with three or more extra interactions was extremely challenging or computationally impossible.

**Figure 5:**
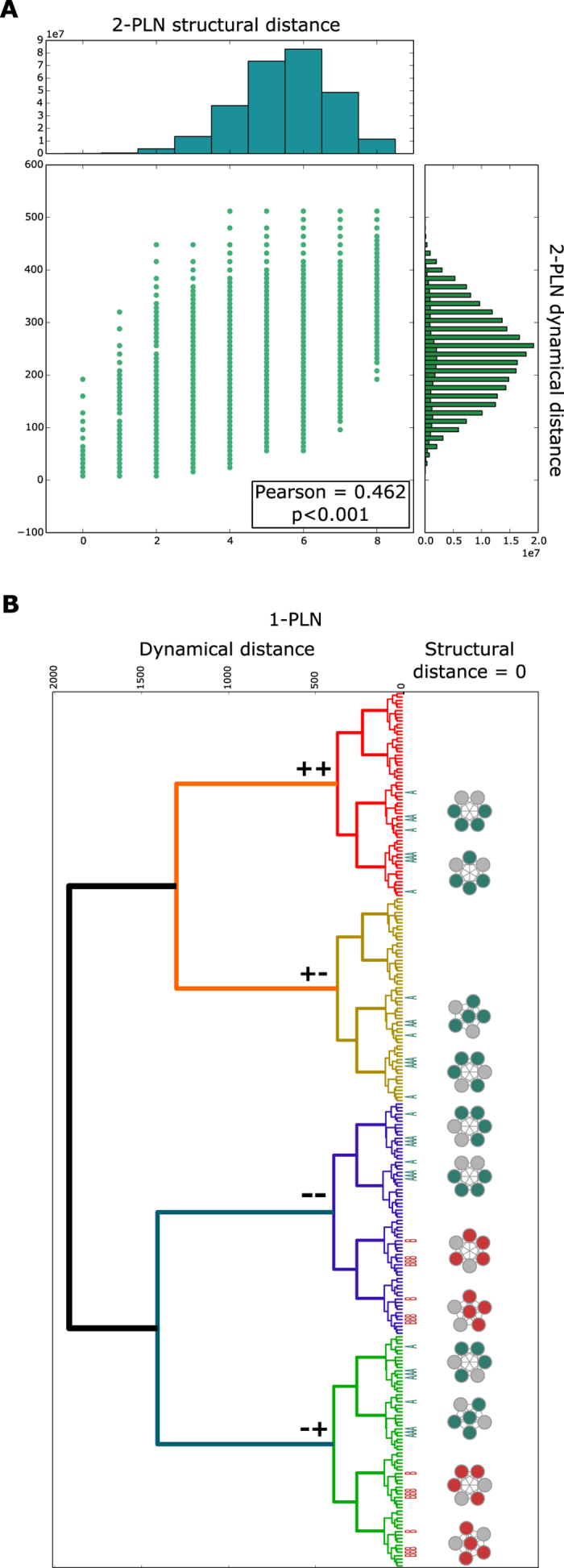
PLNs characterization. (**A**) 2-PLNs relationship between dynamical and structural distance. (**B**) On the left side, 1-PLNs dendogram using the dynamical distance. Each leaf of the dendogram represent a 1-PLN. As observed, 1-PLNs containing the results of the same pathway variant form distinguishable clades, colored with red (++ variant), yellow (+− variant), purple (−− variant) and green (−+ variant). The blue A and red B beside the dendogram represent the two combinations of functionalities found in 1-PLNs and are aligned with the 1-PLNs that contain them. 1-PLNs not aligned with an A or a B do not have any functional circuit. On the right side, clusters formed of 1-PLNs with a structural distance of 0. The blue and red nodes of the clusters are approximately aligned with their corresponding 1-PLNs marked with the blue As and red Bs in the dendogram. Grey nodes of the clusters correspond to PLNs with no functional circuits.

First, we analyzed if structurally similar PLNs followed similar dynamics (Fig. 1C). As observed in Fig. 5A, dynamical and structural distances are weakly correlated. Because the correlation is weak, structurally close PLNs can have large dynamical distances and vice versa. Thus, even when dynamical and structural properties are correlated, this correlation is not sufficient to distinguish between different network strucutres^15^,^32^,^33^,^34^. Then, we noticed that PLNs producing the expected results of the same pathway variant are dynamically closer than networks producing the results of a different pathway variant. Likewise, PLNs with the same combination of functionalities are contained in clusters of PLNs with the same structure. Interestingly, PLNs within the same cluster and with the same combination of functionalities are among the more similar networks at the dynamic level (Fig. 5B). Thus, PLNs with the same combination of functionalities and the same structure form small groups with extremely similar dynamic properties. Based on these results, we believe that studying the characteristics of these regions could be an important step towards a better and more general understanding of molecular regulation and could allow for a better characterization of regulatory networks to improve the scope of traditional methods for the inference of regulatory interactions. The analysis of such regions and their integration in epistasis analysis context remain as interesting questions for future research.

## Conclusions and Discussion

We characterized the effect of adding certain interactions within unidirectional and hierarchical pathway structures. These extra interactions create realistic regulatory structures with a resulting non-unidirectional and non-hierarchical organization containing motifs that we named Pathway-like Networks (PLNs). Additionally, we included certain procedures to ensure the creation of only biologically meaningful dynamics^3^,^22^,^28^. As a result, PLNs have realistic structural and dynamical properties. We used a Boolean formalism to model the dynamic of these networks for many reasons, such as its simplicity and the fact that it have been successfully used to model many gene regulatory networks^10^,^11^,^22^,^23^,^24^. However, Boolean formalisms has some limitations that should be addressed in the future. For example, in certain cases the level of expression or concentration of genes and proteins produces regulatory interactions that cannot be modeled in Boolean terms (e.g., aggravating and synthetic interactions). Despite the limitation of the Boolean formalism, our work clearly shows that PLNs have a great dynamical diversity, characterized by the number and the size of attractors.

The explosion in the dynamical possibilities of the PLNs was expected from previous works. For example, Kauffman^9^ hypothesized that the number of attractors in random Boolean networks increases as 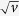. Later, it was found that the number of attractors in Boolean networks increases faster than any power law as the number of variables increases^35^. A strict comparison between these proposals and our results is difficult, because both Samuelsson and Kauffman’s proposals were based on studies with a fixed number of connections, while our networks have a variable connectivity. Nonetheless, our results clearly show that the dynamical diversity of PLNs is vast, compared with the dynamical diversity of unidirectional and hierarchical pathways.

We then focused on the role of network motifs, specially feedback circuits, in the size and the number of attractors. We found that, as the number of positive feedback circuits increases, the number of attractors increases and the size of the attractors decreases. We also observed the exact opposite relation between the number of negative feedback circuits and the number and size of attractors. These results were expected considering that positive and negative feedback circuits are required for multistability and oscillations^12^,^13^. As it has been reported^31^, the positive/negative feedback circuits ratio give the correct trend for the number and the size of the attractors. These results are interesting, but they do not explain how feedback circuits in PLNs, which can have multiple coupled feedback circuits, regulate the size and number of attractor. Thus, we looked for a more mechanistic understanding of the regulation of the size and the number of attractors by analyzing the coupling of feedback circuits. We found that the combination of functionalities (i.e., the way feedback circuits couple) is a key regulator of the number and the size of attractors. In general, PLNs with the same combination of functionalities can produce the same number and size of attractors, independently of its structure.

It is important to note that the same combination of functionalities can produce attractors of different size and number, indicating that the combination of functionalities definition does not capture completely the regulation of the size and the number of attractors, leaving many interesting issues for future research. Some of these issues are, if an extended or modified definition of the combination of functionalities would allow to completely understand how multiple circuits regulate the size and the number of attractors. For example, does considering other properties, such as the functionality of interactions between variables of different circuits could improve our understanding of how circuits regulate the number and the size of attractors? It might also be that the property regulating the size is not the same property that regulates the number of attractors. Another important point for future research is that we compared networks with the same number of variables, but it could be that the same combination of functionalities in a network with different variables will produce attractors with different properties. Hence, we wonder if there is a way to characterize the number and size of the attractors in a network, independently of the number of variables in such a network. Anyhow, we believe that studying how multiple feedback circuits couple and modify the properties of a network, will provide the answer to many of these questions. Thus, it is worthy of further research and could provide insightful information about molecular regulation in general.

In accordance with previous observations^15^,^23^,^36^, we found that several PLNs with different structures are capable of producing the same number, size, and even the same set of attractors. This result emphasizes how limited are the traditional methods for the analysis of experimental results, such as the epistasis analysis^1^,^18^,^19^. First, because such analyses consider only a restricted number of possible networks, they are not well suited to deal with the huge diversity of possible dynamic behaviors in real molecular networks. Second, because such methods are unable to distinguish between alternative networks producing the same set of attractors. Thus, we looked for the number of PLNs that produced the epistasis results. In 2-PLNs, the number of networks that produced the same set attractors was so large, that we needed to constrain our search to a limited number of PLNs structures. It is important to remember that all these networks produce exactly the same attractors, which represent the results expected by epistasis analysis. As a consequence, we can conclude that with the use of epistasis analysis multiple regulatory structures are indistinguishable^16^. Even more, we found that in some cases they can even produce wrong gene regulation inferences^21^. These incorrect inferences are due to the appearance of alternative pathways that can produce the expected behaviors. There may not be general rules to infer complex network structures, such as PLNs^15^,^16^. However, our results suggest that it is fundamental to expand the analyses of regulatory interaction, allowing them to include and consider the presence of regulatory motifs, such as feedback circuits. Considering feedback circuits could be specially relevant, as according to our results, networks sharing the same structure and the same combination of functionalities produce dynamically similar regions. Consequently, in principle, it should be possible to use information, such as the combination of functionalities, to produce better inferences of regulatory structures. In fact, our preliminary studies suggest that not assuming a unidirectional and hierarchical structure, already improves the inference of regulatory structures^37^. Thus, we believe that a more general understanding of the combination of functionalities and its relation with networks structure and dynamic will open possible ways to study and analyze molecular regulation of biological processes.

## Additional Information

**How to cite this article:** Azpeitia, E. *et al*. The combination of the functionalities of feedback circuits is determinant for the attractors’ number and size in pathway-like Boolean networks. *Sci. Rep.* **7**, 42023; doi: 10.1038/srep42023 (2017).

**Publisher's note:** Springer Nature remains neutral with regard to jurisdictional claims in published maps and institutional affiliations.

## Supplementary Material

Supplementary File

## Figures and Tables

**Table 1:** 2-PLNs feedback circuits.

	2-PLNs			
	Total ± CI	Ratio ± CI	Positive ± CI	Negative ± CI
70%				
n−s−	3.34 ± 0.04	1.25 ± 0.02	1.71 ± 0.02	1.62 ± 0.03
n−s+	3.64 ± 0.04	0.74 ± 0.02	0.98 ± 0.02	2.64 ± 0.04
n+s−	3.77 ± 0.04	2.53 ± 0.02	3.13 ± 0.04	0.65 ± 0.02
n+s+	4.72 ± 0.04	1.11 ± 0.02	2.25 ± 0.03	2.45 ± 0.03
80%				
n−s−	3.34 ± 0.04	1.25 ± 0.02	1.71 ± 0.02	1.62 ± 0.03
n−s+	3.70 ± 0.04	0.67 ± 0.02	0.99 ± 0.02	2.70 ± 0.03
n+s−	4.46 ± 0.04	2.71 ± 0.02	3.69 ± 0.04	0.72 ± 0.02
n+s+	5.66 ± 0.05	1.16 ± 0.02	2.87 ± 0.03	2.76 ± 0.03
90%				
n−s−	3.34 ± 0.04	1.25 ± 0.02	1.71 ± 0.02	1.62 ± 0.03
n−s+	3.74 ± 0.04	0.66 ± 0.02	1.02 ± 0.02	2.72 ± 0.03
n+s−	4.71 ± 0.04	2.83 ± 0.02	3.88 ± 0.04	0.71 ± 0.02
n+s+	NA	NA	NA	NA
95%				
n−s−	3.34 ± 0.04	1.25 ± 0.02	1.71 ± 0.02	1.62 ± 0.03
n−s+	4.65 ± 0.04	0.62 ± 0.02	1.31 ± 0.02	3.39 ± 0.04
n+s−	5.08 ± 0.04	3.00 ± 0.02	4.28 ± 0.04	0.73 ± 0.02
n+s+	NA	NA	NA	NA
99%				
n−s−	3.34 ± 0.04	1.25 ± 0.02	1.71 ± 0.02	1.62 ± 0.03
n−s+	5.17 ± 0.05	0.62 ± 0.02	1.51 ± 0.02	3.66 ± 0.04
n+s−	5.51 ± 0.05	3.30 ± 0.02	4.68 ± 0.04	0.76 ± 0.02
n+s+	NA	NA	NA	NA

Positive/negative ratio and number of positive and negative feedback circuits in 2-PLNs divided by category (n+s+, n−s+, n+s− and n−s−) using different thresholds for the number and the mean size of attractors. The number of positive and negative feedback circuits within the same category, have significant differences in all cases (*P* < 0.001). Differences of the total and the ratio between categories is shown in the Supplementary Material. NA stand for not enough data available. The “Total” column corresponds to the sum of positive and negative feedback circuits.

**Table 2:** 1-PLNs feedback circuits.

	1-PLNs			
	Total ± CI	Ratio ± CI	Positive ± CI	Negative ± CI
80%				
n−s−	2.59 ± 0.12	1.06 ± 0.03	1.24 ± 0.08	1.35 ± 0.08
n−s+	2.57 ± 0.07	0.57 ± 0.03	0.29 ± 0.02	2.29 ± 0.07
n+s−	2.74 ± 0.09	2 ± 0	2.46 ± 0.09	0.29 ± 0.03
n+s+	NA	NA	NA	NA
90%				
n−s−	2.59 ± 0.12	1.06 ± 0.03	1.24 ± 0.08	1.35 ± 0.08
n−s+	2.52 ± 0.10	0.75 ± 0.07	0.15 ± 0.02	2.37 ± 0.09
n+s−	2.88 ± 0.13	2 ± 0	2.63 ± 0.12	0.25 ± 0.04
n+s+	NA	NA	NA	NA
95%				
n−s−	2.59 ± 0.12	1.06 ± 0.03	1.24 ± 0.08	1.35 ± 0.08
n−s+	2.67 ± 0.17	NA	0	2.67 ± 0.17
n+s−	3.00 ± 0.00	NA	3.00 ± 0.00	0
n+s+	NA	NA	NA	NA

Positive/negative ratio and number of positive and negative feedback circuits in 1-PLNs divided by category (n+s+, n−s+, n+s− and n−s−) using different thresholds for the number and the mean size of attractors. The number of positive and negative feedback circuits within the same category, have significant differences in all cases (*P* < 0.001 except n−s− for which *P* < 0.057). Differences of the total and the ratio between categories is shown in the Supplementary Material. It was not possible to do statistical analyses with 95% due to the lack of variability. The “Total” column corresponds to the sum of positive and negative feedback circuits. NA stand for not enough data available.
