# Erratum: The combination of the functionalities of feedback circuits is determinant for the attractors’ number and size in pathway-like Boolean networks

**DOI:** 10.1038/srep44964

**Published:** 2017-03-22

**Authors:** Eugenio Azpeitia, Stalin Muñoz, Daniel González-Tokman, Mariana Esther Martínez-Sánchez, Nathan Weinstein, Aurélien Naldi, Elena R. Álvarez-Buylla, David A. Rosenblueth, Luis Mendoza

Scientific Reports 7: Article number: 42023; 10.1038/srep42023 published online: 02 10 2017; updated: 03 22 2017.

This Article contains typographical errors.

In the methods section under subheading ‘**Boolean networks’**, “In Boolean networks, variables can only take one of two possible values, 0 or 1, and their dynamics is described by


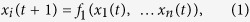


where *x*_*i*_(*t* + 1) represents the value of variable i at the time *t* + 1 as a Boolean function *F*_*i*_ of its n regulators *x*_*i*_(*t*), …, *x*_*n*_(*t*) at the current time”.

should read:

“In Boolean networks, variables can only take one of two possible values, 0 or 1, and their dynamics is described by


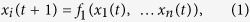


where *x*_*i*_(*t* + 1) represents the value of variable i at the time *t* + 1 as a Boolean function *f*_*i*_ of its n regulators *x*_*i*_(*t*), …, *x*_*n*_(*t*) at the current time”.

In the Results section under subheading ‘**Non-hierarchical and non-unidirectional interactions greatly increases the dynamical diversity of pathways**’,

“Because the inputs follow the identity function, the minimum number of attractors is equal to 2|inputsl|, where *inputs* is the set of inputs”.

should read:

“Because the inputs follow the identity function, the minimum number of attractors is equal to 2^|*inputs*|^ where *inputs* is the set of inputs”.

