# Supplementary material for: The combination of the functionalities of feedback circuits is determinant for the attractors’ number and size in pathway-like Boolean networks
Source: Sci Rep. 2017 Feb 10;7:42023. doi: 10.1038/srep42023 (PMC5301197; doi:10.1038/srep42023)
Supplement: Supplementary File [file srep42023-s1.pdf]

# Supplementary File: The combination of the functionalities of feedback circuits is determinant for the attractors' number and size in pathway-like Boolean networks

**Eugenio Azpeitia[1,*], Stalin Muñoz[2,+], Daniel González-Tokman[3,+], Mariana Esther Martínez-Sánchez[4,5,+], Nathan Weinstein[6,+], Aurélien Naldi[7], Elena R. Álvarez-Buylla[5,8], David A. Rosenblueth[2], and Luis Mendoza[9]**

[1]INRIA project-team Virtual Plants, joint with CIRAD and INRA, Montpellier Cedex 5, France.
[2]Instituto de Investigaciones en Matemáticas Aplicadas y en Sistemas, Universidad Nacional Autónoma de México, Apdo. 20-126, 01000 México, D.F., México.
[3]CONACYT, Instituto de Ecología, A. C., Antiguo camino a Coatepec 351, El Haya, 91070. Xalapa, Veracruz, México.
[4]Programa de Doctorado en Ciencias Biomédicas, Universidad Nacional Autónoma de México, México.
[5]Instituto de Ecología, Universidad Nacional Autónoma de México, México.
[6]ABACUS: Laboratorio de Matemáticas Aplicadas y Cómputo de Alto Rendimiento del Departamento de Matemáticas, Centro de Investigación y de Estudios Avanzados CINVESTAV-IPN, Carretera México-Toluca Km 38.5, La Marquesa, Ocoyoacac, Estado de México, 52740 México.
[7]DIMNP UMR CNRS 5235, Université de Montpellier. France.
[8]Centro de Ciencias de la Complejidad, Universidad Nacional Autónoma de México, México.
[9]Instituto de Investigaciones Biomédicas, Universidad Nacional Autónoma de México, México.
[+]These authors contributed equally to this work.
[*]Corresponding author: eugenio.azpeitia-espinosa@inria.fr

## Supplementary Methods

### *Boolean networks basic definitions*

Let $\mathbb{B} = \{0,1\}$ and $\mathbb{N}_{\leq n} = \{1,2,\ldots,n\}$ an initial segment of natural numbers. We define a *synchronous Boolean network with n components* as a function $f\colon \mathbb{B}^n \to \mathbb{B}^n$. The *i-th component of f* is a function $f_i\colon \mathbb{B}^n \to \mathbb{B}$ such that $f_i(x) = f(x)_i$.

A *state* of the network is a tuple $x = (x_1, x_2, \ldots, x_n)$ such that $x \in \mathbb{B}^n$. The dependency of the state on the discrete time parameter $t$ is denoted as $x(t)$ and obeys the evolution rule given by $f$. That is for all $t \in \mathbb{Z}$

$$x(t+1) = f(x(t))\,. \tag{1}$$

In order to relate a synchronous Boolean network with a molecular network, we interpret each component of a state $x$ as representing the activation state of a particular variable denoting a molecule included in the network. Given a set of variables $V = \{v_1, \ldots, v_n\}$, we define a bijective function $\chi : \mathbb{N}_n^+ \to V$ to relate the components of the state $x$ with their respective variables. The molecule represented by the variable $\chi(i)$ is said to be *active* if $x_i = 1$ and *inactive* otherwise.

For synchronous Boolean networks, an *attractor* is a set of states $A \subseteq \mathbb{B}^n$ where for all $x(0) \in A$ there exist $l > 0$ such that $l = \min_k x(k) = x(0)$ and for all $s \in \mathbb{N}_{\leq l}$, $x(s) \in A$; $l$ is the *size* of the attractor.

For simplicity, we refer to the variable $v$ by its position $i$ in the vector $x$. For a state $x \in \mathbb{B}^n$ and a variable $i$ we denote as $x \sim i$ the vector resulting from replacing the value of $x_i$ with the complement of $x_i$.

Given two variables $i$ and $j$ and the update function of variable $j$, namely $f_j$, *i activates j* if there exists a pair of network states $x$, $y$ that differ only in the state of activation of variable $i$, that is, $y = x \sim i$, $x_i = 0$ and $y_i = 1$, such that $f_j(y) - f_j(x) > 0$. Conversely, *i inhibits j* if there exists a pair of network states $x$, $y$ that differ only in the state of activation of variable $i$, that is, $y = x \sim i$, $x_i = 0$ and $y_i = 1$, such that $f_j(y) - f_j(x) < 0$. An *interaction*, denoted as the pair $(i, j)$, $i, j \in \mathbb{N}_{\leq n}$ is *functional* if variable $i$ activates or inhibits node $j$. Note that according to this definition, it is possible for variable $i$ to both activate and inhibit variable $j$. A non-functional interaction does not provide useful information about the biological system and it is an accepted convention that interactions, where a molecule activates and inhibits the same gene are scarce in molecular

regulations[1,2]. Thus, we excluded both non-functional regulations and regulations where variable $i$ both activate and inhibit variable $j$.

### *Circuits functionality analyses*

A feedback circuit is defined as a set of directed interactions forming a closed path. Feedback circuits can be positive or negative. The sign of a circuit is given by the signs of its interactions. A circuit is positive if it has an even number of negative interactions, it is negative otherwise. It is important to note that the sole presence of a circuit in a network does not guarantee the appearance of the corresponding dynamical behavior (i.e., oscillations or multistability). Thus, a circuit is considered functional if at least one combination of the states of external regulators of its members allows all interactions of the circuit to be functional together[3].

In more formal terms, the *functionality context of the interaction*$(i,j)$ of a Boolean network $f$ is the set $\Phi(f,i,j)$ defined by:

$$\Phi(f,i,j) = \{x \mid f_j(x) \neq f_j(x \sim i) \text{ and } x \in \mathbb{B}^n\} \tag{2}$$

For a Boolean network $f$ we say that $\mathscr{G}_f$ is its *structure* or *interaction graph* $\mathscr{G}_f = \langle V, \mathscr{I}_f^+, \mathscr{I}_f^- \rangle$, where: $\mathscr{I}_f^+$ is its set of *positive interactions* defined by

$$\mathscr{I}_f^+ = \{(\chi(i),\chi(j)) \mid x \in \Phi(f,i,j) \text{ and } \ x_i = f_j(x)\} \tag{3}$$

and $\mathscr{I}_f^-$ is its set of *negative interactions* defined by

$$\mathscr{I}_f^- = \{(\chi(i),\chi(j)) \mid x \in \Phi(f,i,j) \text{ and } \ x_i \neq f_j(x)\} \tag{4}$$

For a circuit $C = (c_1, c_2, \ldots, c_k)$ (simple cycle) with no shortcuts of an interaction graph $\mathscr{G}_f$, where $c_i \in \mathbb{N}_{\leq n}$, $c_i \neq c_j$ if $i \neq j$, we define the *functionality context of the circuit C*, denoted $\Phi(f,C)$ as follows:

$$\Phi(f,C) = \bigcap_{i=1}^{k} \Phi(f, c_i, c_{(i \bmod k)+1}) \,. \tag{5}$$

The circuit $C$ is *functional* if $\Phi(f,C)$ is not empty.

The *functionality context for a circuit C with shortcuts* $S = \{(c_i,c_j) \mid |(j \bmod k) - i| \neq 1\}$ is defined by:

$$\Phi(f,C,S) = \Phi(f,C) - \bigcup_{(i,j)\in S} \{x \mid x \in \Phi(f,C) \text{ and } x \sim i \notin \Phi(f,C)\} \,. \tag{6}$$

As with the previous case the circuit $C$ is functional if $\Phi(f,C,S)$ is not empty.

The *restricted functionality context* of $\Phi(f,C,S)$ by the set of nodes $P = \{\rho_1, \rho_2, \ldots, \rho_k\}$ having a Boolean constant function (i.e. $\forall x, \forall \rho \in P \, f_\rho(x) = c$ and $c \in \mathbb{B}$) is defined by

$$\Phi(f,C,S)[P] = \{x \mid \forall \rho \in P \, x_\rho = f_\rho(x) \text{ and } x \in \Phi(f,C,S)\} \tag{7}$$

A *functional circuit description* is given by the triple $(C,n,\varsigma)$ where $C$ is a circuit, $n = |\Phi(f,C,S)[P]|$ the cardinality of its restricted functionality context, an its sign $\varsigma \in \mathbb{S}$ , where $\mathbb{S} = \{+,-\}$ is the set of signs.

The *combination of the functionality of (feedback) circuits* of a Boolean network $f$ is defined as the set of functional circuit descriptions for all circuits in its interaction graph $\mathscr{G}_f$.

### *Networks structural and dynamical distances*

We define the *adjacency matrix* of a graph $G = \langle V, E \rangle$ with $V = \{v_1, \ldots, v_n\}$ and $E \in V \times V$ as $\mathscr{A}(G) = (a_{ij})$, $(a_{ij}) \in \mathbb{B}^{n\times n}$ with entries satisfying

$$a_{ij} = \begin{cases} 1 & \text{if } (v_i,v_j) \in E \\ 0 & \text{otherwise)} \end{cases} \tag{8}$$

Accordingly, the *structural distance* $D_{\text{str}}(f,g)$ between two Boolean networks $f$ and $g$ is defined by

$$D_{\text{str}}(f,g) = \left\| \mathscr{A}(\langle V, \mathscr{I}_f^+ \cup \mathscr{I}_f^- \rangle) - \mathscr{A}(\langle V, \mathscr{I}_g^+ \cup \mathscr{I}_g^- \rangle) \right\|_1 \tag{9}$$

where $\|\cdot\|_p$ is the matrix entrywise norm defined by

$$\|(a_{ij})\|_p = \left(\sum_i \sum_j |a_{ij}|^p\right)^{1/p} \tag{10}$$

The *signed structural distance* $D^{\varsigma}_{\text{str}}(f,g)$ between two Boolean networks $f$ and $g$ is defined by

$$D^{\varsigma}_{\text{str}}(f,g) = \sum_{\varsigma_1 \in \mathbb{S}} \sum_{\varsigma_2 \in \mathbb{S}} \#_{\varsigma_1} \cdot \#_{\varsigma_2} \cdot \left\| \mathscr{A}(\langle V, \mathscr{I}^{\varsigma_1}_f \rangle) - \mathscr{A}(\langle V, \mathscr{I}^{\varsigma_2}_g \rangle) \right\|_1 \tag{11}$$

where $\# : \mathbb{S} \to \{-1, 1\}$, is defined by

$$\#_{\varsigma} = \begin{cases} -1 & \text{if } \varsigma = - \\ 1 & \text{if } \varsigma = + \end{cases} \tag{12}$$

The *dynamical distance* between two Boolean networks $f$and$g$ is defined by

$$D_{\text{dyn}}(f,g) = \sum_{x \in \mathbb{B}^n} \sum_{i \in \mathbb{N}_{\leq n}} |f_i(x) - g_i(x)| \tag{13}$$

We compared each pair of networks $A$ and $B$ of size $N$ using the three distances described above, implementing the necessary algorithms in Python. The dynamical distance clustering analysis was done using the scipy/linkage function (ward)[4] for the dynamical distance. We considered that only distances below a certain threshold where valid edges (0 and 8 for the structural and dynamical distance, respectively). In the resulting networks each node represents a PLN and the edges' weight corresponds to the dynamical or structural distance. We analyzed the network properties using python/networkx[5].

### *PLNs simulations, analyses and queries*

For section 2.1 and 2.2 PLNs were simulated using R/BoolNet 2.1.1[6]. There were $9 \times 10^3$ biologically meaningful 1-PLNs, all of which were analyzed. As for biologically meaningful 2-PLNs, due to their astronomical number, we used samples of sizes between $10^3$ and $32.8 \times 10^6$ of them to analyze their properties. The combinations of functionalities were analyzed using GINsim[7].

For the sections 2.4 and 2.5, the search of PLNs with the epistasis expected set of attractors (see section 3.3) was done with Griffin[8]. Griffin is a software tool that uses symbolic algorithms for the inference of Boolean networks. Griffin transforms the set of constraints into a Boolean sentence, which in turn using a Tseitin transformation[9,10] is converted into an equisatisfiable conjunctive normal form sentence. This sentence is then provided as an input to a SAT solver, SAT4j[11]. When the solver finds an assignment of the Boolean variables that make the sentence true, this assignment is returned to Griffin. Griffin then decodes the assignment into a set of Boolean functions corresponding to the network dynamics.

Certain biological constraints were added to Griffin to formulate the epistasis queries. First, we use a set of *generalized interactions* which are a set of gene interaction constraints that corresponded to the MUS,OUS and MP interactions. The expected fixed point attractors of the 2-PLNs required partially defined fixed points and the double mutant experiment required multiple genes mutations with partially defined states. All of these biological constraints were transformed by Griffin into the Boolean sentence representing the query. Finally, we prohibited networks that exhibit cyclic trajectories in the state space. As it is computationally intractable to add this constraint a priori, Griffin performs a posteriori refinement of cycles using Dubrova and Teslenko's SAT based algorithm[12]. Any satisfying assignment will be decoded to a biologically meaningful Boolean network.

All the data generated via simulations, analyses and queries are available under request.

### *Statistical Analyses*

All statistical analyses were carried out in R version 3.2.3[13].

To test the relationship between number of attractors and attractor average size we carried out a non-parametric Spearman rank correlation, given that the assumptions of parametric correlation were violated (Fligner-Killeen test for homogeneity of variances $X^2 = 526.784$, $d.f. = 9$, $P < 0.001$ ).

The differences between the circuits and structural properties in the singles and 2-PLNs were analyzed with generalized linear models (GLM) with Poisson error structure and log link function. In GLMs with overdispersion (overdispersion test $P < 0.05$;[14]) we used models with quasipoisson errors[15]. For analyzing attractor sizes and ratio of positive/negative circuits we used generalized least squares (GLS) to account for heterogeneous variances found by type (Fligner-Killeen test $P < 0.001$)[16,17].

Finally, in order to test the frequency distribution of networks ($N = 6.3X10^7$ networks) of the attractors size and number we used Kolmogorov-Smirnov tests for log-normal, exponential, normal and Poisson distributions implemented in R package nortest. To test for power law distributions we used a bootstrapping procedure with 30 simulations in R package poweRlaw[18].

## Statistical analyses results

### *Attractors properties*

The attractors size was significantly larger for 2-PLNs than for 1-PLNs (GLS $F1,18998 = 217.63$,$P < 0.001$; $C.I.95\%$: 2-PLNs= $1.62 \pm 0.012$; 1-PLNs = $1.49 \pm 0.012$).

The number of attractors was significantly larger for 2-PLNs than for 1-PLNs (Poisson GLM $z = 103.2$, $d.f. = 1,18998$, $P < 0.001$; $C.I.95\%$: 2PLN = $6.22 \pm 0.049$; 1-PLNs = $2.91 \pm 0.035$).

### *Feedback circuits and PLNs structure*

The number of combinations was significantly higher in 2-PLNs than in n–s– 2-PLNs (Poisson GLM: $z = 104.6$, $P < 0.001$, 2-PLNs $1,510 \pm 11.07$, n–s– 2-PLNs $788 \pm 7.74$).

The number of combinations of functionalities contained in each structure was significantly larger for 2-PLN than for n–s– 2-PLN (Quasipoisson GLM $t = 53.96$, $d.f. = 30768$, $P < 0.001$; $C.I.95\%$: 2PLN = $14.24 \pm 0.199$; n–s– 2-PLNs = $2.36 \pm 0.146$).

The mean number of combinations of functionalities contained in each structure was not significantly different for 1-PLNs and 1-n–s– PLNs (Quasipoisson GLM $t = 1.423$, $d.f. = 19$, $P = 0.172$).

The mean number of structures containing the same combination of functionalities was similar between 2-PLNs and n–s– 2-PLNs (Quasipoisson GLM $Res.Dev. = 280865$, $d.f. = 254468$, $P = 0.331$, 2-PLNs $1.46 \pm 0.010$, n–s– 2-PLNs $1.43 \pm 0.044$ ).

### *Relations*

In 1-PLNs, the relationship between the number of attractors and the attractors average size was significant and negative (Poisson GLM: $Res.Dev. = 1139.8$, $d.f. = 8998$, $z = -22.59$, $P < 0.001$). In 1-PLNs, the relationship between the number of attractors and the number of negative feedback circuits was significant and negative (Poisson GLM: $Res.Dev. = 5683.8$, $d.f. = 8998$, $z = -30.45$, $P < 0.001$). The relationship between the number of attractors and the number of positive feedback circuits was significant and positive (Poisson GLM: $Res.Dev. = 5633.5$, $d.f. = 8998$, $z = 34.29$, $P < 0.001$). The relationship between the attractors average size and the number of negative feedback circuits was significant and positive (Poisson GLM: $Res.Dev. = 5428.5$, $d.f. = 8998$, $z = 38.15$, $P < 0.001$). The relationship between the attractors average size and the number of positive feedback circuits was significant and negative (Poisson GLM: $Res.Dev. = 32122$, $d.f. = 8998$, $z = -37.45$, $P < 0.001$).

In 2-PLNs to test the relationship between the number of attractors and the attractors average size we took a random sample of 10,000 networks and carried out a generalized linear model (GLM) with Poisson errors and log link function. We used power tests in G*Power software (version 3.1.9.2) to confirm that this number of sampled networks was enough to achieve high power values in the analyses (higher than 0.90)[19]. In particular, we used two-tailed z-tests for Poisson distributions, using alpha values of 0.05 including the exponential of each model's coefficients as input parameters. When the independent variable was a count (number of attractors) we specified the observed lambda and when the predictor variable was continuous (attractor?s size) we used the observed mean and standard deviation[20,21]. For the relation between positive loops and attractor size we needed to sample 30,000 networks in order to achieve a power of 0.90. We found a significant, negative relation between the number of attractors and the attractors average size (Poisson GLM: $Res.Dev. = 1968.9$, $d.f. = 9998$, $z = -14.00$,$P < 0.001$). The relationship between the number of attractors and the number of negative feedback circuits was significant and negative (Poisson GLM: $Res.Dev. = 146.46$, $d.f. = 9998$, $z = -11.79$, $P < 0.001$). The relationship between the number of attractors and the number of positive feedback circuits was significant and positive (Poisson GLM: $Res.Dev. = 4975.5$, $d.f. = 9998$, $z = 39.60$, $P < 0.001$). The relationship between the attractors average size and the number of negative feedback circuits was significant and positive (Poisson GLM: $Res.Dev. = 9607.10$, $d.f. = 9998$, $z = 39.45$, $P < 0.001$). The relationship between the attractors average size and the number of positive feedback circuits was significant and negative (Poisson GLM: $Res.Dev. = 32122$, $d.f. = 29998$, $z = -19.36$, $P < 0.001$).

For 1-PLN, the connectivity was positively related with the number of attractors ($PoissonGLMz = 3.25$, $P = 0.001$), but not with the size of attractors ($LM : F1,8989 = 0.206$, $P = 0.650$). For 2-PLN, the connectivity was positively related with the number of attractors ($PoissonGLMz = 41.27$, $P < 0.001$), and with the size of attractors ($LM : F1,15936 = 1089.1$, $P < 0.001$).

### *Distributions*

For the number of attractors, the frequency distribution differed significantly from a normal distribution ($D = 0.205$, $P < 0.001$) or from a Poisson distribution ($D = 0.256$, $P < 0.001$). For the attractors size, the frequency distribution differed significantly from a normal distribution ($D = 0.265$, $P < 0.001$), from a Poisson distribution ($D = 0.420$, $P < 0.001$) or from a power law distribution ($KS = 0.013$, $Xmin = 6$, $Scaling = 10.278$, $P < 0.001$). For the number of attractors, the frequency distribution differed significantly from a log-normal distribution ($D = 0.853$, $P < 0.001$) and from an exponential distribution ($D = 0.474$, $P < 0.001$). The attractors size frequency distribution also differed significantly from a log-normal ($D = 0.307$, $P < 0.001$) and an exponential distribution ($D = 0.401$, $P < 0.001$).

## References


1. Raeymaekers, L. Dynamics of boolean networks controlled by biologically meaningful functions. *Journal of Theoretical Biology* **218**, 331–341 (2002).
2. Azpeitia, E., Benítez, M., Padilla-Longoria, P., Espinosa-Soto, C. & Alvarez-Buylla, E. R. Dynamic network-based epistasis analysis: boolean examples. *Frontiers in Plant Sciences* **15**, 2:92 (2010).
3. Naldi, A., Thieffry, D. & Chaouiya, C. *Decision Diagrams for the Representation and Analysis of Logical Models of Genetic Networks*, vol. 4695 of *Computational Methods in Systems Biology. Lecture Notes in Computer Science*, 233–247 (Springer Berlin Heidelberg, 2007).
4. Müllner, D. fastcluster: Fast hierarchical, agglomerative clustering routines for r and python. *Journal of Statistical Software* **53**, 1–18 (2013).
5. Hagberg, A., Schult, D. & Swart, P. Networkx reference (2012).
6. Müssel, C., Hopfensitz, M. & Kestler, H. A. Boolnet – an R package for generation, reconstruction and analysis of Boolean networks. *Bioinformatics* **26**, 1378–1380 (2010).
7. Chaouiya, C., Naldi, A. & Thieffry, D. *Logical Modelling of Gene Regulatory Networks with GINsim*, 463–479 (Springer New York, New York, NY, 2012).
8. Rosenblueth, D. A., Muñoz, S., Carrillo, M. & Azpeitia, E. Inference of Boolean Networks from Gene Interaction Graphs Using a SAT Solver. In *Algorithms for Computational Biology*, 235–246 (Springer International Publishing, 2014).
9. Prestwich, S. D. CNF encodings. *Handbook of Satisfiability* **185**, 75–97 (2009).
10. Tseitin, G. S. On the complexity of derivation in propositional calculus. In *Automation of reasoning*, 466–483 (Springer, 1983).
11. Le Berre, D. & Parrain, A. The sat4j library, release 2.2, system description. *Journal on Satisfiability, Boolean Modeling and Computation* **7**, 59–64 (2010).
12. Dubrova, E. & Teslenko, M. A SAT-based algorithm for finding attractors in synchronous Boolean networks. *IEEE/ACM Transactions on Computational Biology and Bioinformatics (TCBB)* **8**, 1393–1399 (2011).
13. R Development Core Team. *R: a Language and Environment for Statistical Computing* (Austria, 2015).
14. Kleiber, C. & Zeileis, A. *Applied Econometrics with R* (New York, U.S.A, 2008).
15. Crawley, M. J. *The R Book* (London, U.K., 2007), 1st edn.
16. Venables, W. N. & Ripley, B. D. *Modern Applied Statistics with S* (New York, U.S.A, 2002), fourth edn.
17. Zuur, A. F., Ieno, E. N., Walker, A. A., N. J.and Saveliev & Smith, G. M. *Mixed Effects Models and Extensions in Ecology with R* (New York, U.S.A, 2009).
18. Gillespie, C. S. Fitting heavy tailed distributions: The poweRlaw package. *Journal of Statistical Software* **64**, 1–16 (2015). URL http://www.jstatsoft.org/v64/i02/.
19. Ellis, P. D. *The Essential Guide to Effect Sizes* (Cambridge University Press, 2010).
20. Faul, F., Erdfelder, E., Lang, A.-G. & Buchner, A. G*power 3: A flexible statistical power analysis program for the social, behavioral, and biomedical sciences. *Behavior Research Methods* **39**, 175–191 (2007).
21. Faul, F., Erdfelder, E., Buchner, A. & Lang, A.-G. Statistical power analyses using g*power 3.1: Tests for correlation and regression analyses. *Behavior Research Methods* **41**, 1149–1160 (2009).

**Table S1. 2-PLNs contrasts the ratio and total number of feedback circuits between the different groups** Results from linear models (circuits ratio) and generalized linear model with Poisson distribution (total circuits). In the upper part of the diagonal for each percentage used are p-values for circuits ratio and in the lower part of the diagonal are p-values for total circuits, corresponding to Table 1.

| 2-PLNs | | | | | |
|---|---|---|---|---|---|
| Percentage | PLN group | n–s– | n–s+ | n+s– | n+s+ |
| 70% | n–s– | — | $< 0.001$ | $< 0.001$ | $< 0.001$ |
| | n–s+ | $< 0.001$ | — | $< 0.001$ | $< 0.001$ |
| | n+s– | $< 0.001$ | $< 0.001$ | — | $< 0.001$ |
| | n+s+ | $< 0.001$ | $< 0.001$ | $< 0.001$ | — |
| 80% | n–s– | — | $< 0.001$ | $< 0.001$ | $< 0.001$ |
| | n–s+ | $< 0.001$ | — | $< 0.001$ | $< 0.001$ |
| | n+s– | $< 0.001$ | $< 0.001$ | — | $< 0.001$ |
| | n+s+ | $< 0.001$ | $< 0.001$ | $< 0.001$ | — |
| 90% | n–s– | — | $< 0.001$ | $< 0.001$ | NA |
| | n–s+ | $< 0.001$ | — | $< 0.001$ | NA |
| | n+s– | $< 0.001$ | $< 0.001$ | — | NA |
| | n+s+ | NA | NA | NA | — |
| 95% | n–s– | — | $< 0.001$ | $< 0.001$ | NA |
| | n–s+ | $< 0.001$ | — | $< 0.001$ | NA |
| | n+s– | $< 0.001$ | $< 0.001$ | — | NA |
| | n+s+ | NA | NA | NA | — |
| 99% | n–s– | — | $< 0.001$ | $< 0.001$ | NA |
| | n–s+ | $< 0.001$ | — | $< 0.001$ | NA |
| | n+s– | $< 0.001$ | $< 0.001$ | — | NA |
| | n+s+ | NA | NA | NA | — |

**Table S2. 1-PLNs contrasts of the ratio and total number of feedback circuits between the different groups** In the upper part of the diagonal are p-values of contrasts from linear models for circuits ratio (even when variances were heterogeneous in some cases, modifying variance structure with GLS did not improve model fit); in the lower part of the diagonal are p-values of contrasts from Poisson GLMs for total circuits, corresponding to Table 1. 70, 95 and 99% results are not presented due to the lack of data. NA=data not available to carry out contrasts.

| 1-PLNs | | | | | |
|---|---|---|---|---|---|
| Percentage | PLN group | n–s– | n–s+ | n+s– | n+s+ |
| 80% | n–s– | — | $< 0.001$ | $< 0.001$ | NA |
| | n–s+ | 0.812 | — | $< 0.001$ | NA |
| | n+s– | 0.044 | 0.003 | — | NA |
| | n+s+ | NA | NA | NA | — |
| 90% | n–s– | — | $< 0.001$ | $< 0.001$ | NA |
| | n–s+ | 0.372 | — | $< 0.001$ | NA |
| | n+s– | 0.002 | $< 0.001$ | — | NA |
| | n+s+ | NA | NA | NA | — |

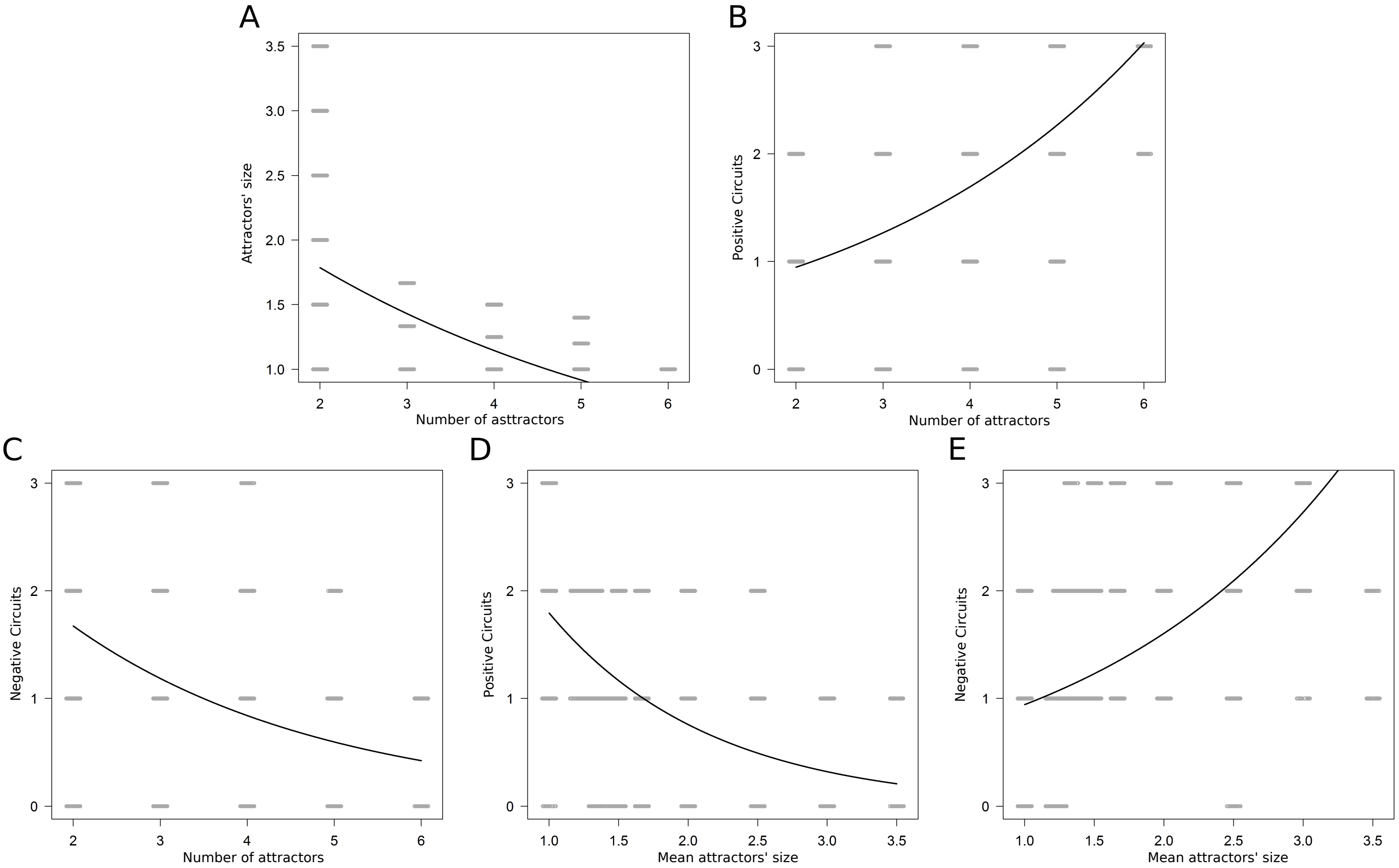


**Figure S1. 1-PLNs properties**.(A) Number of attractor vs. attractors mean size. (B) and (C) number of attractors vs. quantity of negative and positive feedback circuits, respectively. (D) and (E) size of attractors vs. quantity of negative and positive feedback circuits, respectively. As observed, negative and positive feedback circuits have opposite effects, just as in 2-PLNs. Each point represents a single 2-PLN data. Points are displaced in the X axis only for visual purpose. The lines are predicted by Poisson GLMs.

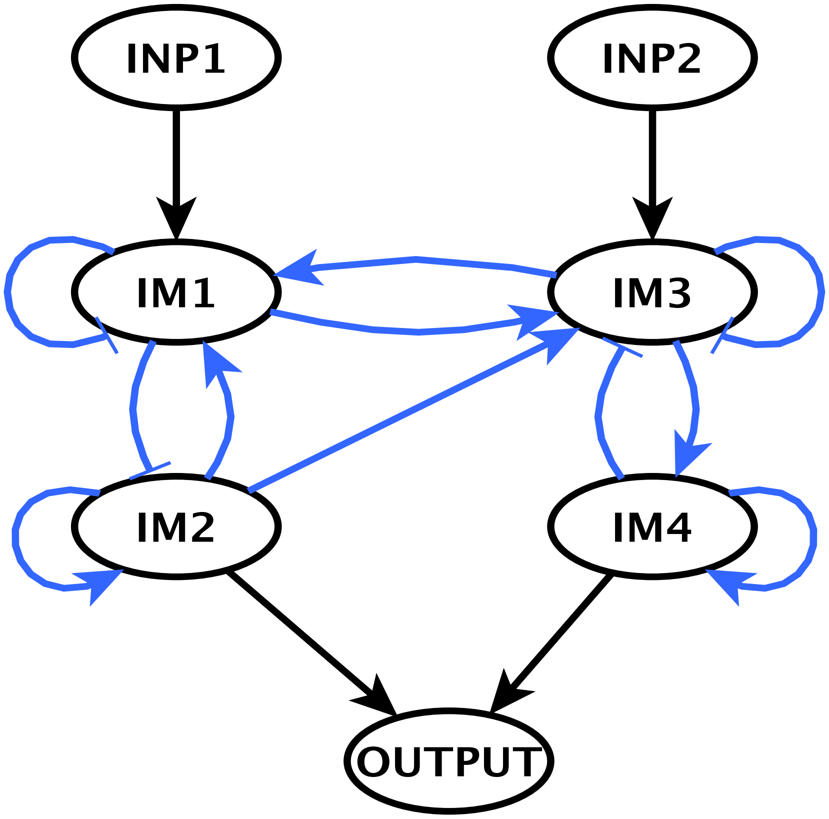


**IM1 = (INP1 | IM2 | ! IM1) & IM3**
**IM2 = ! IM1 & IM2**
**IM3 = INP2 & IM1 & IM2 & ! IM3 & ! IM4**
**IM4 = IM3 | IM4**
**OUTPUT = IM4 & IM2**

**Figure S2. Example of a n+s– 2-PLN with an low positive/negative feedback circuits ratio**. Interaction graph of a 2-PLN that has three positive and five negative feedback circuits formed with the blue interactions. Because, this PLN has more negative (all functional) than positive feedback circuit (two of them functional), its positive/negative ratio (0.6 considering all feedback circuits and 0.4 considering only the functional ones) is lower than the mean feedback circuits ratio on n–s+ 2-PLNs using any percentage. Thus, we would not expect a n+s– behavior. Anyhow, this network produces 16 fixed-point attractors (i.e., it is a n+s– 2-PLN) using the Boolean functions below (the inputs follow the identity function). &, | and ! stand for the AND, OR and NOT logical operators.

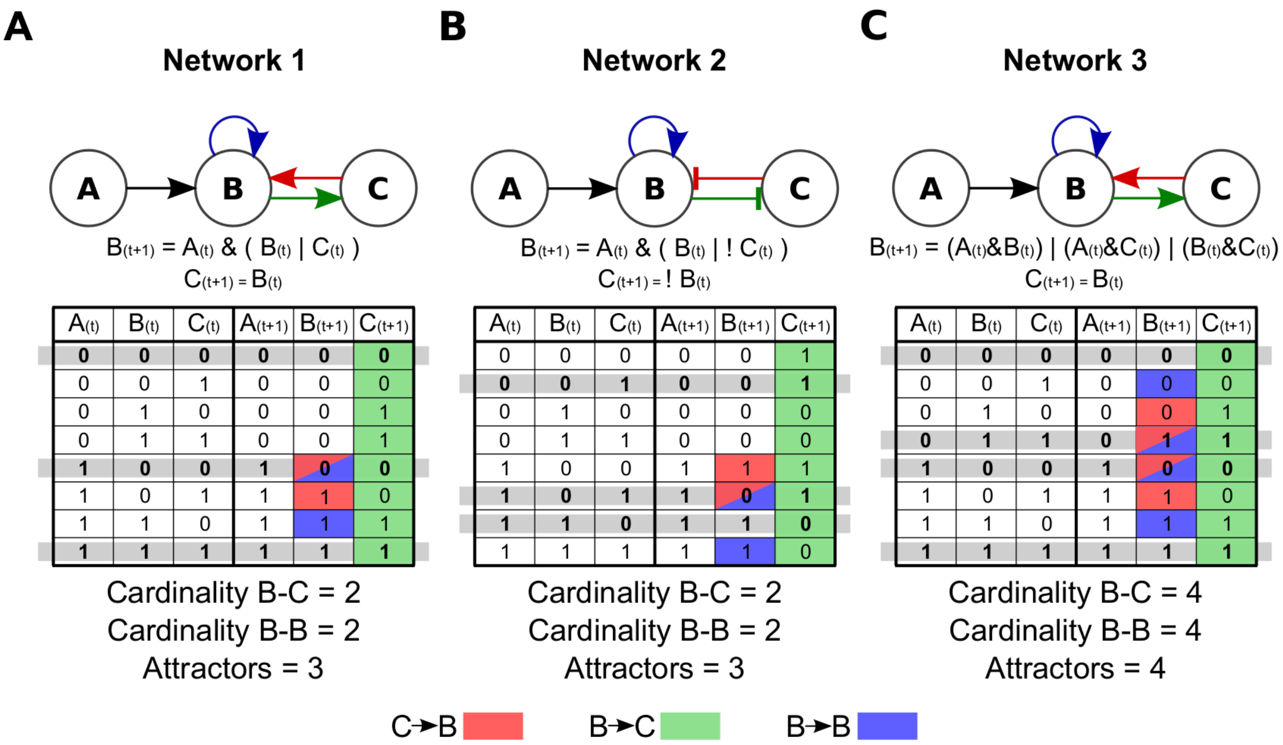


Network 1

| $A_{(t)}$ | $B_{(t)}$ | $C_{(t)}$ | $A_{(t+1)}$ | $B_{(t+1)}$ | $C_{(t+1)}$ |
|---|---|---|---|---|---|
| **0** | **0** | **0** | **0** | **0** | **0** |
| 0 | 0 | 1 | 0 | 0 | 0 |
| 0 | 1 | 0 | 0 | 0 | 1 |
| 0 | 1 | 1 | 0 | 0 | 1 |
| **1** | **0** | **0** | **1** | **0** | **0** |
| 1 | 0 | 1 | 1 | 1 | 0 |
| 1 | 1 | 0 | 1 | 1 | 1 |
| **1** | **1** | **1** | **1** | **1** | **1** |

Network 2

| $A_{(t)}$ | $B_{(t)}$ | $C_{(t)}$ | $A_{(t+1)}$ | $B_{(t+1)}$ | $C_{(t+1)}$ |
|---|---|---|---|---|---|
| 0 | 0 | 0 | 0 | 0 | 1 |
| **0** | **0** | **1** | **0** | **0** | **1** |
| 0 | 1 | 0 | 0 | 0 | 0 |
| 0 | 1 | 1 | 0 | 0 | 0 |
| 1 | 0 | 0 | 1 | 1 | 1 |
| **1** | **0** | **1** | **1** | **0** | **1** |
| **1** | **1** | **0** | **1** | **1** | **0** |
| 1 | 1 | 1 | 1 | 1 | 0 |

Network 3

| $A_{(t)}$ | $B_{(t)}$ | $C_{(t)}$ | $A_{(t+1)}$ | $B_{(t+1)}$ | $C_{(t+1)}$ |
|---|---|---|---|---|---|
| **0** | **0** | **0** | **0** | **0** | **0** |
| 0 | 0 | 1 | 0 | 0 | 0 |
| 0 | 1 | 0 | 0 | 0 | 1 |
| **0** | **1** | **1** | **0** | **1** | **1** |
| **1** | **0** | **0** | **1** | **0** | **0** |
| 1 | 0 | 1 | 1 | 1 | 0 |
| 1 | 1 | 0 | 1 | 1 | 1 |
| **1** | **1** | **1** | **1** | **1** | **1** |

**Figure S3. Some properties of the combinations of functionalities**. (A-C) Above, the interaction graphs of three networks with two positive feedback circuits. The red, green and blue arrows highlight the interactions forming the circuits. In the middle, the Boolean functions used to solve these networks (*A* follows the identity function). Below, the state diagrams of the networks. The red, green and blue color, highlight the lines where the interactions are functional in the state diagram. The attractors are highlighted with grey. (A) and (B) Network 1 and 2 have the same combination of functionalites comprising two positive feedback circuits, each with a cardinality of 2. (C) Network 3 has a different combination of functionalites of two positive feedback circuits, each with a cardinality of 4. Notice that the same network structure (Network 1 and network 3) can produce different combinations of functionalities, and that different network structures (network 1 and network 2) can produce the same combination of functionalities. Observe that because both circuits share B variable, a change in the cardinality of one circuit can modify the cardinality of the other circuit. Finally, remark that the networks with the same combination of functionality produce the same number of attractors, while networks with different combinations of functionalities produce a different number of attractors. Similar cases exist for the size of the attractors. &, | and ! stand for the AND, OR and NOT logical operators.

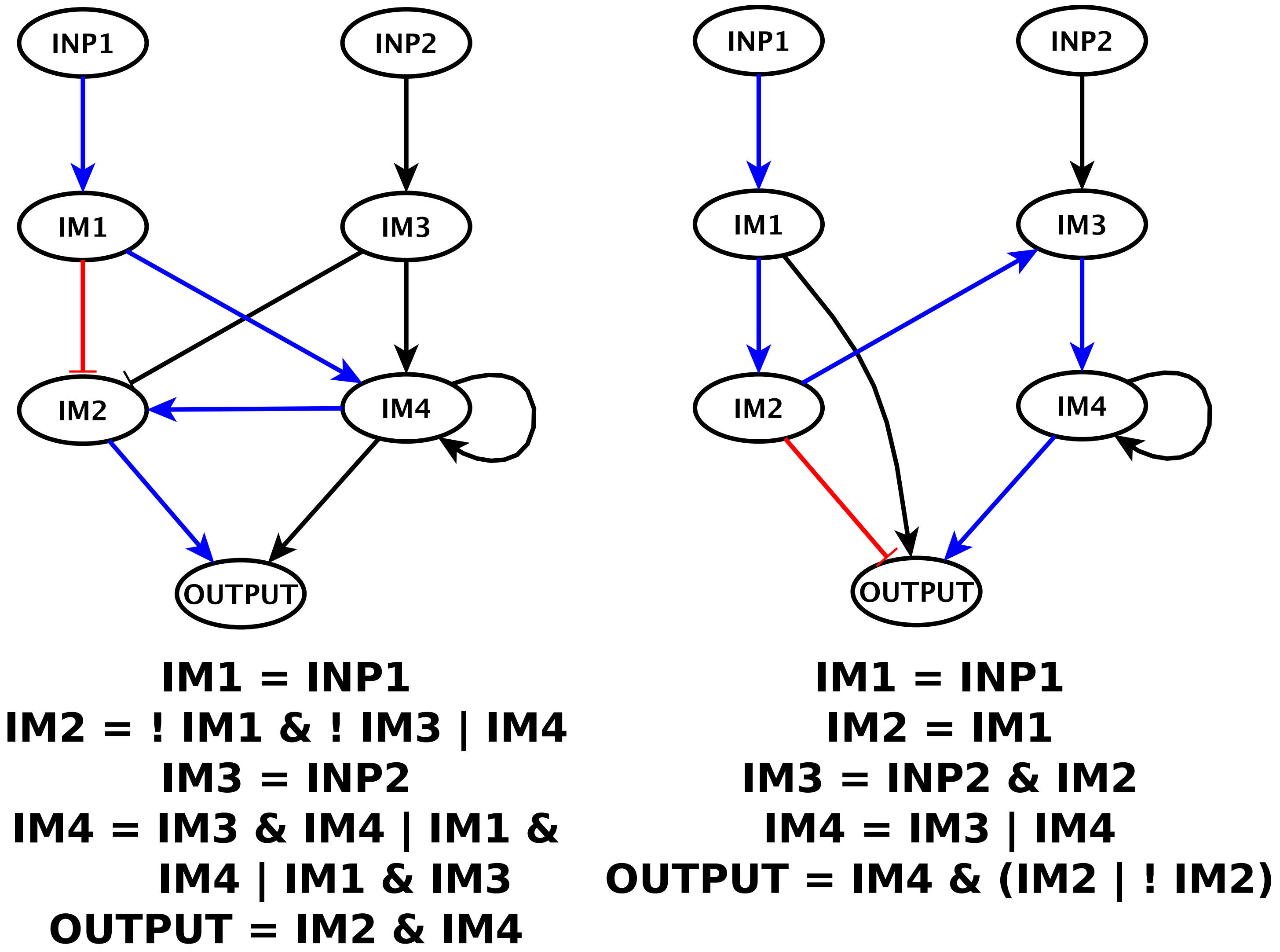


**Figure S4. Two examples of wrongly inferred interactions using epistasis analysis**. Examples of the cases where the interaction from IM1 to IM2 (A) and the interaction from IM2 to OUTPUT (B) are wrongly inferred. In both cases the expected pathway variant is ++. The orange edge is the incorrect inferred interaction and the blue edges are alternative pathways that contains the expected signs of interactions between INP1, IM1, IM2 and OUTPUT with some extra intermediary interactions. Below the Boolean functions for each of these PLNs (the inputs value is fixed to 1). &, | and ! stand for the AND, OR and NOT logical operators.

A

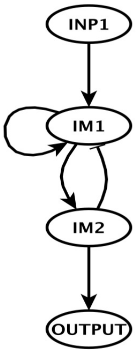


| INP1(t) | IM1(t) | IM2(t) | OUT(t) | INP1(t+1) | IM1(t+1) | IM2(t+1) | OUT(t+1) |
|---|---|---|---|---|---|---|---|
| 1 | 0 | 0 | 0 | 1 | 1 | 0 | 0 |
| 1 | 0 | 0 | 1 | 1 | 1 | 0 | 0 |
| 1 | 0 | 1 | 0 | 1 | 0 | 0 | 1 |
| 1 | 0 | 1 | 1 | 1 | 0 | 0 | 1 |
| 1 | 1 | 0 | 0 | 1 | 1 | 1 | 0 |
| 1 | 1 | 0 | 1 | 1 | 1 | 1 | 0 |
| 1 | 1 | 1 | 0 | 1 | 1 | 1 | 1 |
| 1 | 1 | 1 | 1 | 1 | 1 | 1 | 1 |

B

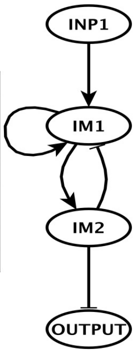


| INP1(t) | IM1(t) | IM2(t) | OUT(t) | INP1(t+1) | IM1(t+1) | IM2(t+1) | OUT(t+1) |
|---|---|---|---|---|---|---|---|
| 1 | 0 | 0 | 0 | 1 | 1 | 0 | 1 |
| 1 | 0 | 0 | 1 | 1 | 1 | 0 | 1 |
| 1 | 0 | 1 | 0 | 1 | 0 | 0 | 0 |
| 1 | 0 | 1 | 1 | 1 | 0 | 0 | 0 |
| 1 | 1 | 0 | 0 | 1 | 1 | 1 | 1 |
| 1 | 1 | 0 | 1 | 1 | 1 | 1 | 1 |
| 1 | 1 | 1 | 0 | 1 | 1 | 1 | 0 |
| 1 | 1 | 1 | 1 | 1 | 1 | 1 | 0 |

C

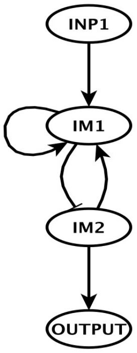


| INP1(t) | IM1(t) | IM2(t) | OUT(t) | INP1(t+1) | IM1(t+1) | IM2(t+1) | OUT(t+1) |
|---|---|---|---|---|---|---|---|
| 1 | 0 | 0 | 0 | 1 | 0 | 1 | 0 |
| 1 | 0 | 0 | 1 | 1 | 0 | 1 | 0 |
| 1 | 0 | 1 | 0 | 1 | 1 | 1 | 1 |
| 1 | 0 | 1 | 1 | 1 | 1 | 1 | 1 |
| 1 | 1 | 0 | 0 | 1 | 1 | 0 | 0 |
| 1 | 1 | 0 | 1 | 1 | 1 | 0 | 0 |
| 1 | 1 | 1 | 0 | 1 | 1 | 0 | 1 |
| 1 | 1 | 1 | 1 | 1 | 1 | 0 | 1 |

D

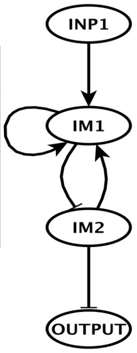


| INP1(t) | IM1(t) | IM2(t) | OUT(t) | INP1(t+1) | IM1(t+1) | IM2(t+1) | OUT(t+1) |
|---|---|---|---|---|---|---|---|
| 1 | 0 | 0 | 0 | 1 | 0 | 1 | 1 |
| 1 | 0 | 0 | 1 | 1 | 0 | 1 | 1 |
| 1 | 0 | 1 | 0 | 1 | 1 | 1 | 0 |
| 1 | 0 | 1 | 1 | 1 | 1 | 1 | 0 |
| 1 | 1 | 0 | 0 | 1 | 1 | 0 | 1 |
| 1 | 1 | 0 | 1 | 1 | 1 | 0 | 1 |
| 1 | 1 | 1 | 0 | 1 | 1 | 0 | 0 |
| 1 | 1 | 1 | 1 | 1 | 1 | 0 | 0 |

IM2 → IM1   IM1 → IM1   IM1 → IM2

**Figure S5. Examples of 1-PLNs with the same combination but different pathway variant**. 1-PLNs interaction graphs containing and producing the expected attractors of the (A) ++, (B) +–, (C) –+ and (D) – – pathway variants. Interestingly, all these 1-PLNs have the same combination of functionalities, comprising a positive feedback circuit from IM1 to IM1 with a cardinality of four, and a negative feedback circuit between IM1 and IM2 with a cardinality of four. In the state diagrames, green, blue and pink colors, highlight the lines where the interactions are functional. The attractors are highlighted with grey.
